# Survey on computational 3D visual optical art design

**DOI:** 10.1186/s42492-022-00126-z

**Published:** 2022-12-19

**Authors:** Kang Wu, Xiao-Ming Fu, Renjie Chen, Ligang Liu

**Affiliations:** School of Mathematics, University of Science and Technology of China, Hefei 230026, China

**Keywords:** Computational art, 3D visual art, Automatic art design, Optical art

## Abstract

Visual arts refer to art experienced primarily through vision. 3D visual optical art is one of them. Artists use their rich imagination and experience to combine light and objects to give viewers an unforgettable visual experience. However, the design process involves much trial and error; therefore, it is often very time-consuming. This has prompted many researchers to focus on proposing various algorithms to simplify the complicated design processes and help artists quickly realize the arts in their minds. To help computer graphics researchers interested in creating 3D visual optical art, we first classify and review relevant studies, then extract a general framework for solving 3D visual optical art design problems, and finally propose possible directions for future research.

## Introduction

3D visual optical art refers to those 3D objects that can produce special effects under a specified light source. These highly diverse arts include sculpture, architecture, and ceramics. They are created to stimulate us through a visual experience. Generally, when artists design 3D visual optical art, they first imagine what they want to show in their minds and then use tools to manufacture it. However, it is undoubtedly a challenge for artists to successfully design complicated 3D visual optical arts, such as anamorphic art and 3D illusion art, and produce satisfactory results. This is because they must go through trial and error to obtain artwork that meets the requirements.

These difficulties encountered by artists have caught the attention of researchers, who are attempting to propose automatic methods to solve various problems for designing 3D visual optical art. Moreover, since artworks often need to be created in the real world, the fabrication problem of 3D visual optical art is also studied. In our view, these design problems are all reconstruction and fabrication problems. Thus, the algorithm first reconstructs the geometry and texture of the object according to the desired visual effect (usually represented as input images) and then fabricates it. Since many methods aim to reduce the errors between the digital models and the fabrication results, this survey focuses on the model reconstruction problems. We find that the model reconstruction formulations are very different, whether the art form is of the same type or not. Then, a unique solution is proposed for each model reconstruction problem, thereby lacking a unified framework. This lack of a general solution framework makes it difficult to model 3D visual optical art at a high level, thereby preventing the development of this field.

The motivation for this work is three-fold. First, this paper can help graphics researchers interested in the field of visual arts quickly learn about related works in this field. Second, with the rise of new techniques, such as neural networks and differentiable rendering, in recent years, researchers have more ways to solve previously intractable problems. Reviewing previous work can help researchers who want to create 3D visual optical arts overcome previous challenges with the new techniques. Third, no 3D visual optical art design survey has been conducted yet. Researchers need a holistic analysis and vision of this field for the future.

This paper reviews 65 related studies during the last ten years and divides them into four categories according to the types of art: (1) shadow, (2) reflection, (3) refraction, and (4) others. At the end of this paper, we find that the existing 3D visual optical art design problems can be reformulated as inverse problems. Then, we propose a general solution framework using the latest graphics technology. Besides, we reveal the current research trends and provide an outlook for future research direction and solutions. We hope our work can help researchers quickly learn about this field and provide them with possible future research directions.

## Shadow

In 3D visual optical art, a class of artwork can only be appreciated when its shadow is formed under a specific light source. We divide these into two categories: (1) shadow art and (2) self-shadowing images. In the first category, the artworks cast their shadows onto walls or other shadow receivers to form a pattern. In the second category, the self-shadow of the artwork forms a pattern. This category differs from the previous one in that the receiver is the object. The object is usually a surface with microstructures. These microstructures are used to produce shadows when placed under a light source.

### Shadow art

Placing 3D objects under a light source to make their shadows form specific patterns is called shadow art. Creating a shadow pattern under one light source is not difficult because the silhouette of a 3D object determines the shape of its shadow. Using existing graphics algorithms [1] can help the artist quickly achieve his goal. However, more complex shadow art, such as soft shadow art and multi-image shadow art, is difficult to design. Researchers propose various algorithms to address the design problems of these shadow arts. We will introduce them in this section.

#### Sculpture

Mitra and Pauly [2] proposed a method for creating a 3D volume sculpture whose shadows show different patterns under different light sources. The main difficulty here is that there may not exist a 3D shape that satisfies all shadow constraints. Their solution is to deform the input binary images until a consistent configuration is obtained. In each iteration, they chose some inconsistent pixels in one shadow image and projected each point in their corresponding rays to other image planes to calculate the distance to the boundary of the shadow pattern. Then, they took the sum of these distances as the cost and chose the point that had the minimum cost. Other input images would be deformed to the projection pixel of this point by using the as-rigid-as-possible algorithm [3].

**Fig. 1:**
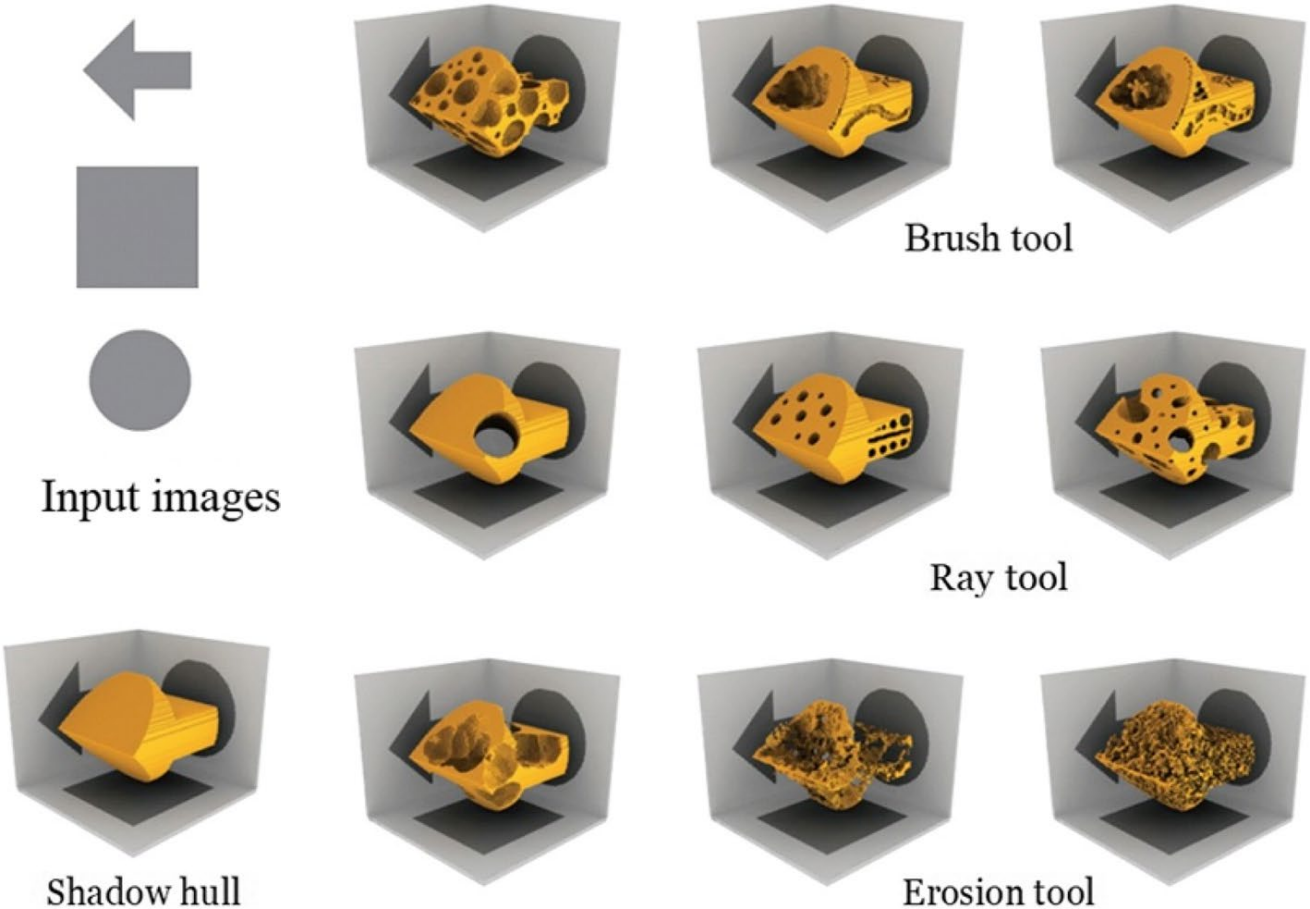
Mitra and Pauly [2] designed some tools to assist the user in modifying the sculpture without altering the desired shadow images

Since each deformation operation reduces inconsistent pixels, their algorithm guarantees a result of satisfying all shadow constraints. For convenience, Mitra and Pauly also used their algorithm to create an interactive 3D editing toolbox for the artist to design shadow art (Fig. 1). Their algorithm allows users to edit the sculpture freely while keeping the shadow images intact. Their algorithm can also deform the input images, which do not lead to a consistent configuration, to get a feasible result. However, their algorithm has no theoretical way to guarantee the deformation bounds, which means that the image distortions may be too significant for extreme cases.

This year, Sadekar et al. [4] used differentiable rendering to solve this problem directly. Differentiable rendering allows the user to obtain the gradient from the image to a 3D object. Therefore, they can solve this problem directly by applying a gradient descent algorithm to the image error function. Their results are better than that of Mitra and Pauly [2] in some cases. However, since they do not process the input images, the result may be poor when the input images do not have consistent configurations.

**Fig. 2:**
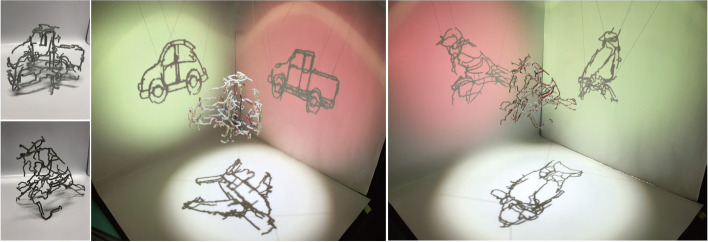
Two results generated by the method of Hsiao et al.[5]

Similar to the work of Mitra and Pauly, Hsiao et al.[5] presented their algorithm for creating multi-view wire sculptures. Unlike Mitra and Pauly’s work, the inconsistent pixels in wire images cannot be eliminated by deforming images. Moreover, in consideration of fabrication, the result should be simply connected. Thus, their goal is to find a 3D path to connect all isolated parts while minimizing the image error between the wire sculpture’s shadows and the input images. They formulated this problem as a conventional minimum spanning tree problem and used a graph to represent all isolated components of the wire sculpture and their spatial relationships. They used 3D printing technology to produce their results to validate the produced art pieces (Fig. 2). Their system can generate smooth continuous wire sculptures, but their algorithm inevitably has noticeable artifacts, which are not very pleasing.

#### Multiple-layered

In this visual art optical class, the artist uses multi-layered objects to generate shadows. We call them multiple-layered for short. The artist can present more wonderful artistic effects due to the higher degrees of freedom of the multi-layer structure. However, designing this visual art class is also more difficult. Light casts shadows after passing through all layers; therefore, modifying any layer affects the result, making it hard for the artist to find a suitable solution.

Hiding a secret image in distributed and shared images (referred to as shares) is one of these visual arts, also called visual cryptography (VC). The shares are translucent, and their patterns can be meaningless, noisy, or meaningful. The user can see the intimate images by physically superimposing the shares. However, decrypting multiple secret images using a unique general share is difficult with traditional VC. To solve this problem, Kita and Miyata [6] proposed an approach. They studied how to hide two secret images, ${I}_{{S}_{1}}$ and ${I}_{{S}_{2}}$, in three images, ${I}_{1}$, ${I}_{c}$, and ${I}_{2}$. They assumed that all input images were binary images. Thus, their goal was to assign white or black to the corresponding output share images, ${S}_{1}$, ${S}_{c}$, and ${S}_{2}$. They also set output images with twice the width and height of the input. Therefore, each $2\times 2$ pixel block corresponded to a pixel in input images. In shares, they denoted the pixel block with two black pixels as a white pixel and three black pixels as a black pixel. While in intimate images, they set the pixel block with one white pixel as a white pixel and no white pixel as a black pixel. Therefore, they could calculate all the pixel combinations in shares and intimate images. These combinations were stored in the look-up table (LUT). The color of each pixel in output images was then set by randomly selecting a valid combination from the LUT.

**Fig. 3:**
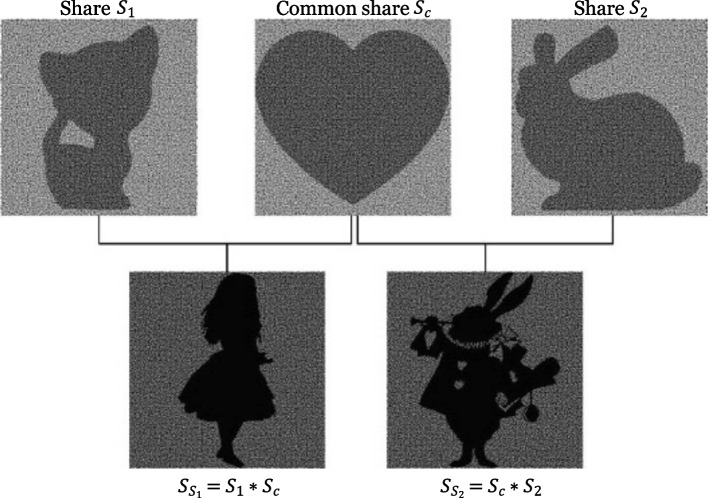
The algorithm proposed by Meghrajani and Mazumdar [8] can produce VC with a unique general share. By superimposing the common share (${S}_{c}$) and other shares (${S}_{1}$*,*${S}_{2}$), the user can see different secret images (${S}_{{S}_{1}}$*,*${S}_{{S}_{2}}$)

Their method can also be extended to a color-extended VC scheme (EVCS). The only thing that needs to be done is to decompose the given images into C-, M-, and Y-channel images and then apply Ostromoukhov’s error diffusion method [7] to convert the images to halftone images. Nevertheless, their output images have low contrast (Fig. 3). Until now, color EVCS remains a challenging and open problem.

Meghrajani and Mazumdar [8] also studied this design problem. They focused on providing threshold security. Their algorithm used a random universal share-generating function when producing a distinct universal share for each secret image. This random universal share-generating function is from the proposed base universal share. Their generated images have a strong security effect. Furthermore, the shares generated by their algorithm can reconstruct lossless secret images.

**Fig. 4:**
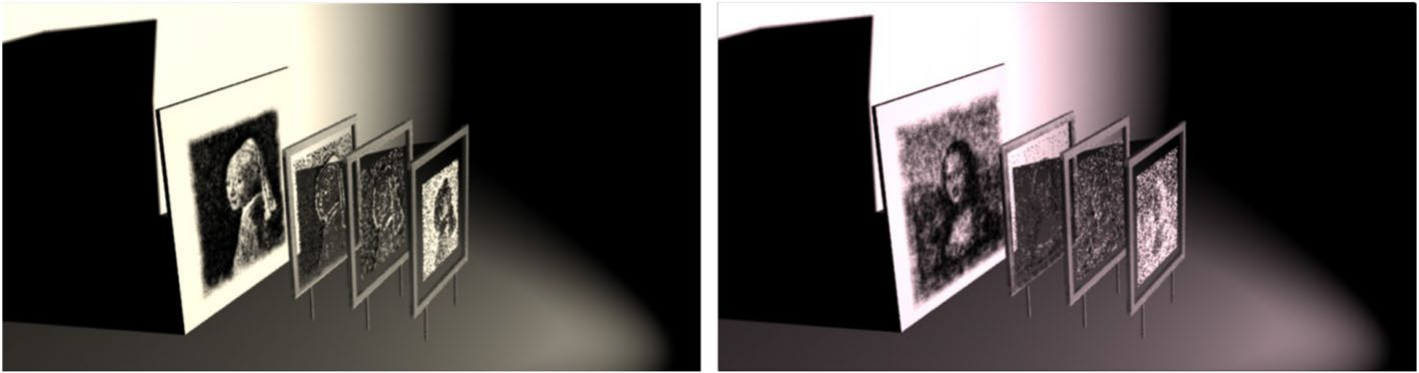
Each layer of the object casts its shadow onto the receiver to form the target image [9]

Another type of multiple-layered shadow art consists of an area light (or an array of point lights) and multiple layers of occluder cells (Fig. 4). It is different from shadow art, in which the 3D object usually casts a binary shadow image under a single point light. Min et al. [9] studied this problem. The key challenge is to arrange the occluders properly so that the shadows they cast are closest to the input image. They formulated the problem as a combinatorial optimization problem with many binary variables. Then, they used a stochastic algorithm to solve it. Their results look promising. However, they did not model the soft shadows finely. We think there is still much space for exploration in soft shadows.

**Fig. 5:**
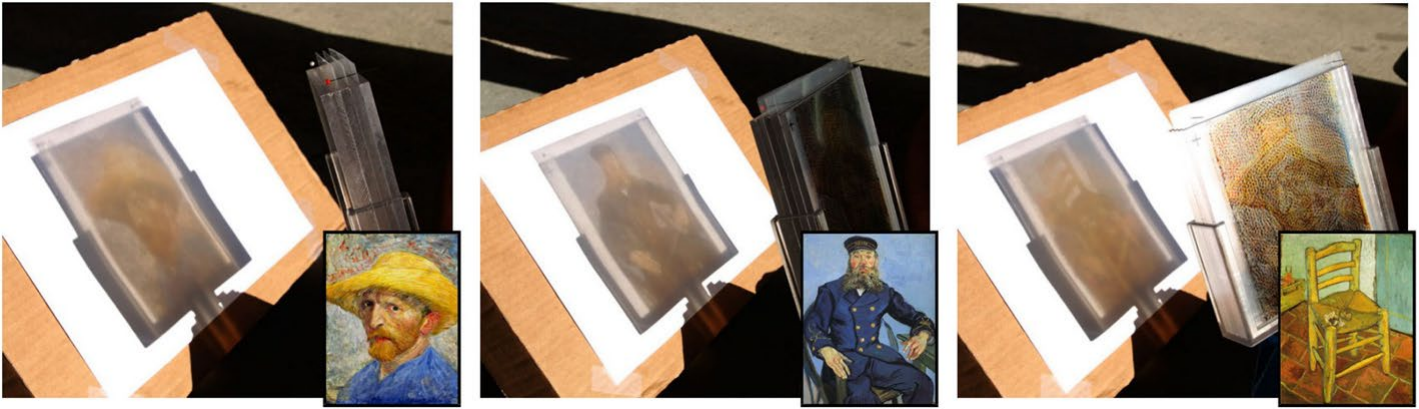
Baran et al. [10] used translucent plates to fabricate shadow artworks that cast different color shadow images when placed at different orientations under sunlight

The artist also uses transparent materials to cast color shadow patterns. Baran et al. [10] presented a method to generate such visual art. Their results consist of multiple layers of translucent plates. When placed under specific light sources, it can cast different color shadow images (Fig. 5).

They simplified the problem as follows. The color of each light ray reaching the receiver is the product of the colors of the intersection points in each layer. The color at each point in the receiver is the sum of the colors of each ray reaching this point. However, this optimization problem is still hard to solve. They chose to only optimize one of the attenuator layers in each iteration. In this case, the objective is convex quadratic. A sparse quadratic programming solver could then be applied to solve it. The speed of their algorithm is relatively fast, but there is no guarantee that the image error is reduced to an acceptable threshold, and the contrast of the output images is not high.

#### Others

Apart from sculpture, buildings are also used to make shadow art. Xiong et al. [11] studied the design problem of architecture shadow art. The challenge is to optimize the building’s shape so that its shadows best match the input patterns while satisfying the topological, functional, and structural requirements. They developed a shape metric to evaluate the result and used an improved cuckoo search metaheuristic to solve the optimization problem. Since they do not modify the input image, their algorithm can only output the closest result if the input images do not have a consistent configuration.

Using human bodies to create shadow art is a more difficult task as there are more constraints. The first challenge is that each character’s pose should be stable, balanced, and plausible. The second is the completeness of the resulting shadows, as we cannot deform the human body into arbitrary shapes. The last challenge is that some shadow patterns require multiple actors to participate, and the artist needs to arrange them reasonably.

To address this problem, Won and Lee [12] introduced their algorithm. They used body shape meshes derived from a 3D skeletal posture to represent actors. Their energy function consists of six formulas. Formula 1 is the visual error between the projected and target shadow contours. Formula 2 penalizes the area difference. Formula 3 encourages the projected shadow to consist of as few parts as possible.

Formula 4 asks the actors to look at the screen as much as possible. Formula 5 guarantees that the poses are reasonable. Formula 6 favors the result as close as possible to the user’s desired poses. Since this energy function is non-differentiable, they chose the covariance matrix adaptation evolutionary strategies algorithm [13] to solve the optimization problem.

Won and Lee also extended their algorithm to character animation. They used the algorithm mentioned above to generate principal poses. Later, this pose was used as the initial value for optimizing neighboring frames. Smoothness, regularization, and contact terms were used to ensure a suitable result during the optimization process. Their algorithm can make the optimization converge to a plausible solution. However, since they used linear skinning and inaccurate body modeling, the results generated in the real world had significant artifacts. Furthermore, although they restricted the model’s range of movements, the results still have some highly difficult acrobatic poses. In the future, more realistic body models or deep learning methods may improve their algorithm.

**Fig. 6:**
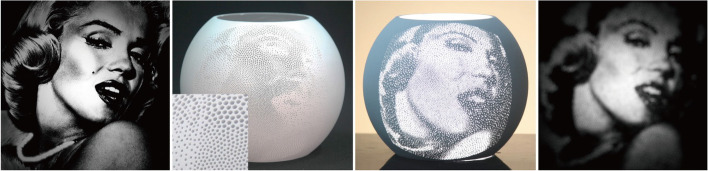
The radii and orientation of the tubes affect the grayscale of the shadow, Zhao et al. [14] take advantage of this fact to make the lampshade into a shadow artwork

In addition, Zhao et al. [14] chose to use lampshades to create shadow art. The generated lampshade can cast continuous grayscale images because the distribution, radii, and orientation of tubes affect the shadow’s grayscale. Their goal is to find a suitable configuration for the tubes. To address this issue, they first defined a suitable density function and calculated a capacity-constrained Voronoi tessellation over the tube. Then, a tube was embedded inside the maximal inscribed circle of each tessellation cell. Their results are not purely binary images but have more details (Fig. 6). Compared with Min et al. [9], although they only used a single-layer structure, their algorithm models the relationship between light intensity and tube configuration well; therefore, their algorithm can generate more refined results.

### Self-shadowing images

Sometimes, the artist uses self-occlusion shadows cast by the 3D object to form patterns. In this case, the shadow receiver becomes the object itself. In this paper, we call this class of visual art self-shadowing images. Creating a self-shadowing image is more complex than shadow art, as the shadow receiver is the object itself. The shadow it casts and receives will change when we modify the object’s shape.

To overcome this challenge, Bermano et al. [15] built ‘walls’ to cast shadows on a plane to create a self-shadowing image. They offered two different methods for solving this problem.

**Fig. 7:**
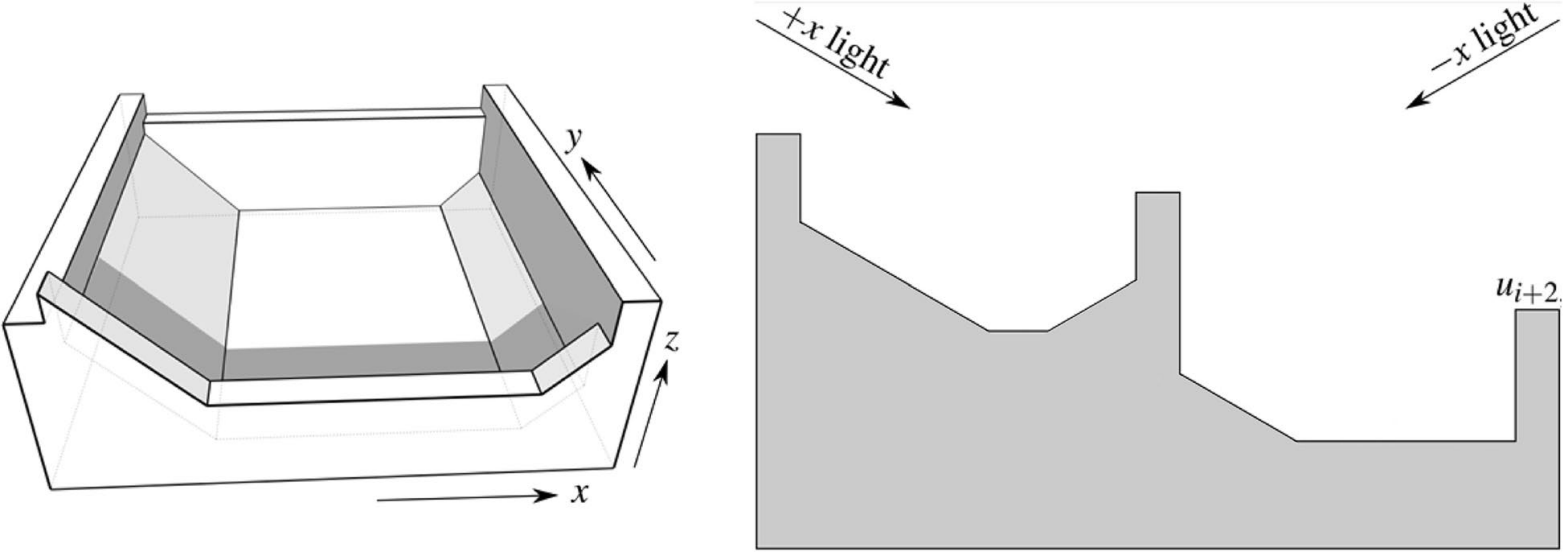
The local method [15] uses walls and chamfers to create shadows. Each grid corresponds to one pixel in input images. The light source direction, when projected on a xy plane, is parallel to the pixels

The local method uses a grid corresponding to the pixels of the input images to generate self-shadowing images. To simplify the problem, the light directions are parallel to the pixel when projected to the xy plane. The height of walls affects each grid’s shaded area, corresponding to the grayscale value of the pixel. Inequality constraints are proposed to ensure that other parts of the grid do not cast shadows and that the shadows are only cast into the neighbor grid. However, they found that the result sometimes was too high to be usable. To address this issue, chamfers are added to the edge of each shadow receiver (Fig. 7). The chamfers are always parallel to the opposite light directions. Therefore, it is only lit by the light from other directions and is brighter than the bottom of the grid because the light directions are more perpendicular to it. When one receiver is too high, adding the chamfers will allow it to be lower while keeping the brightness unchanged. The local method can display three images under different light sources.

**Fig. 8:**
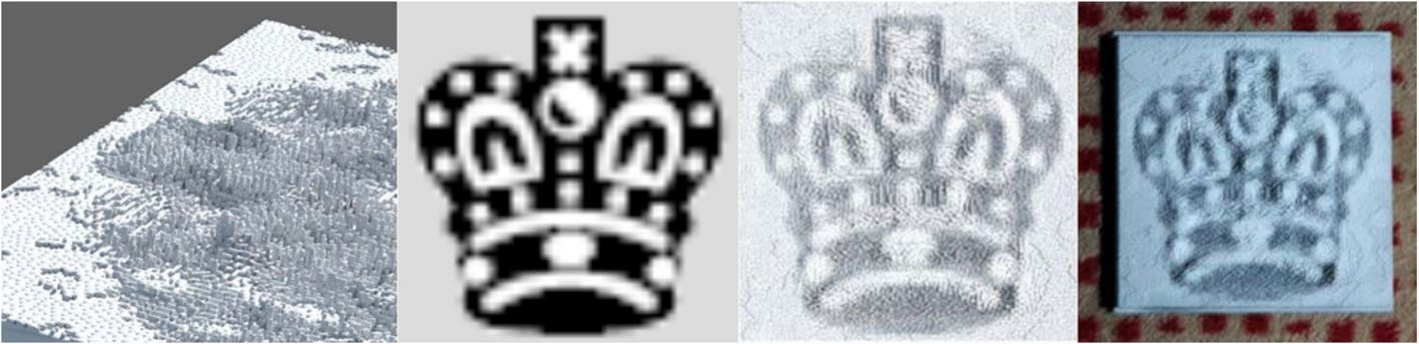
From left to right are the result generated by the global method [15], the input image, the simulated result, and the manufactured result

The global method uses heightfield to produce shadows. However, the height of each element cannot be changed continuously. The shadow cast by each element covers an integer number of pixels precisely. Therefore, each element is either shadowed or not. Their objective function consists of three parts. The first term is the visual difference between the target grayscale and binary images. They used a convolution with a low-pass filter to model the human visual system. The second is the gradient error for preserving edges. The third is the smoothness term for the heightfield. For this method, they could potentially use any number of input images and light in any direction. They used a 3D printer to manufacture their results (Fig. 8).

The results of their local method have distinct striped artifacts caused by reflections from the top of the walls they use to cast shadows. Although their global method does not have this problem, it can only generate binary images. Besides, since they used microstructures to cast shadows, the resolution of the results generated by the two methods was limited.

**Fig. 9:**
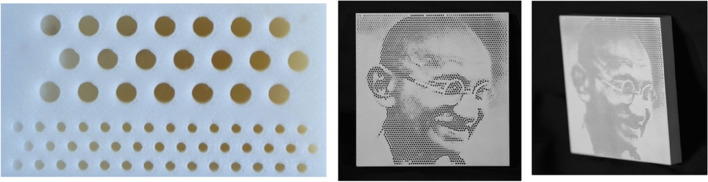
On the left is a close-up of the 3D printing result of Alexa and Matusik [16]. Increasing the depth of the hole causes it to appear darker. They use this fact to create self-shadowing images

Alexa and Matusik [16] chose to make holes in a plane to create self-shadowing images (Fig. 9). Their work is based on a simple observation. The depth of the hole can affect the radiance. They first measured the relation between hole depths and albedo and then used this relationship to optimize the position of holes to match the input image better. However, their method cannot handle more than one image.

## Reflection

Reflection is another way for artists to create 3D visual optical art. It gives the artist a tool to control light and create more visual effects. Generally, the artist’s use of reflection falls into two categories: (1) specular and (2) diffuse.

In the specular category, mirrors are chosen to provide other views of the 3D object. These views are often very different from what the user sees directly. This contrast gives the user a novel visual experience. However, adding mirrors to 3D visual optical art requires the artist to calculate the light path, which means he needs to know the corresponding knowledge of geometry and physics. It undoubtedly increases the difficulty of design. While in the diffuse category, the artist modifies the reflectance of a 3D object to give it a peculiar appearance under certain light sources. Likewise, designing the reflectance of an object is not an easy task.

Over the past decade, many researchers proposed algorithms for these design problems. In this section, we divide these works into three categories: (1) optimizing mirror, (2) optimizing object, and (3) optimizing reflectance.

### Optimizing mirror

Mirrors are one of the essential components in the reflection arts. By optimizing the position and shape of the mirror, the artist can control the path of the reflected light and what the user sees in the mirror. The former allows the artist to project reflected light onto the receiver to form a pattern, while the latter can deform the object by using the mirror to achieve the artist’s desired result.

#### Reflecting light sources

**Fig. 10:**
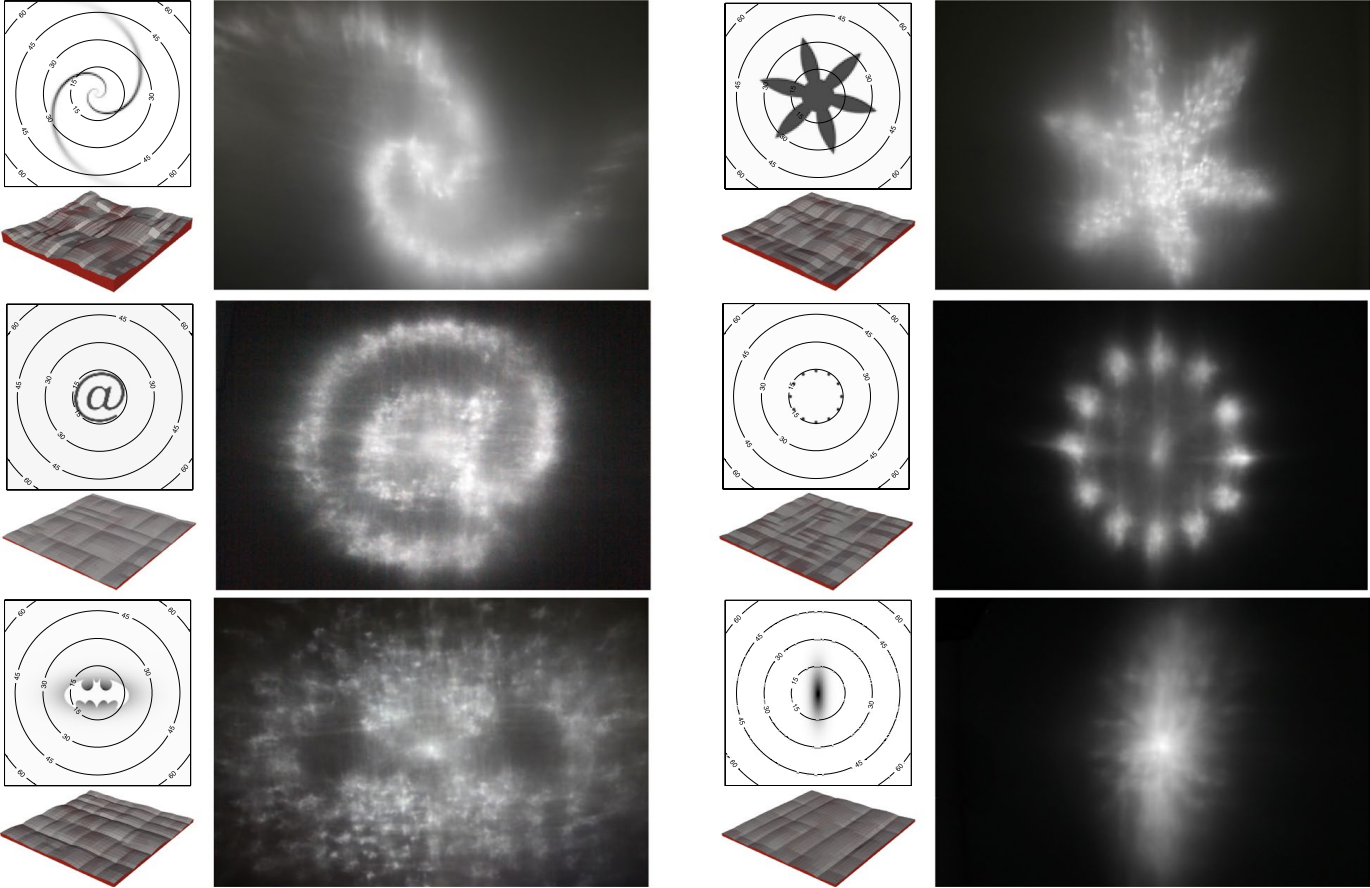
Six results of Weyrich et al.[17] manufactured using CNC. Top-left is the target distribution. Bottom-left is the object and right is the reflected image

A study by Weyrich et al. [17] shows how to manufacture a mirror heightfield to reflect light to form the desired pattern. To simplify the problem, they made the following assumptions. The slope of the microfacets was restricted to 65 degrees so that shadowing, masking, and interreflections were not considered. They also assumed that the base material had a spatially homogeneous bidirectional reflectance distribution function (BRDF). Under the above assumptions, each element’s BRDF is a rotation of the base BRDF according to its normal, and the aggregate BRDF is the sum of these BRDF. The half-angle/difference-angle parameterization proposed by Rusinkiewicz [18] was then used to derive the net effect. Once the user inputs the desired highlight shape, their algorithm first constructs a half-angle distribution from the distribution of reflected intensities and then converts the half-angles distribution into a microfacet (normal) distribution. These problems can be formulated as deconvolution problems. Weyrich et al. used the iterative Lucy-Richardson deconvolution algorithm [19, 20] to solve it. The half-angle component of the base BRDF was used as the convolution kernel. After that, the next step of their algorithm is to transform the microfacet distribution to a height-field. They sampled the microfacet distribution to get a discrete set of microfacet orientations and optimized the heightfield to generate a *C*^*0*^ surface. Their final result was fabricated by using a desktop Computer Numerical Control (CNC). The results proved their algorithm (Fig. 10). The main limitation of their method is that the resolution at which they sampled the microfacet distribution is too low. Thus, their output images are discontinuous. Moreover, they cannot control the brightness of each microfacet’s reflection. These cause their algorithm’s results not to perform well enough in some cases.

The work of Mas et al.[21] is closer to a practical application than art. They need to generate a reflector shape that produces a light distribution as close as possible to a user-provided one. The difference from the work of Weyrich et al. is that the input to this problem is a lighting area rather than a pattern. Besides, light may bounce multiple times inside their reflector. They defined the reflector parametrically and used a binary tree to search for the best value in the parametric space to better approximate the desired light distribution. Each node of the tree represents a reflector. At each construction step of the tree, they utilized the statistical information about tree nodes to propose a heuristic method and used this method to choose a previously created node. Once chosen, the selected node was split into two subranges and replaced by two new child nodes. Which parameter range to split was determined by an ancestors-based heuristic method. Their proposed method has been tested in a real case study based on European road lighting safety regulations. The disadvantage of their algorithm is that the solution speed is not fast enough, and since they use a search algorithm, the relevant parameters significantly influence the results.

#### Reflecting objects

**Fig. 11:**
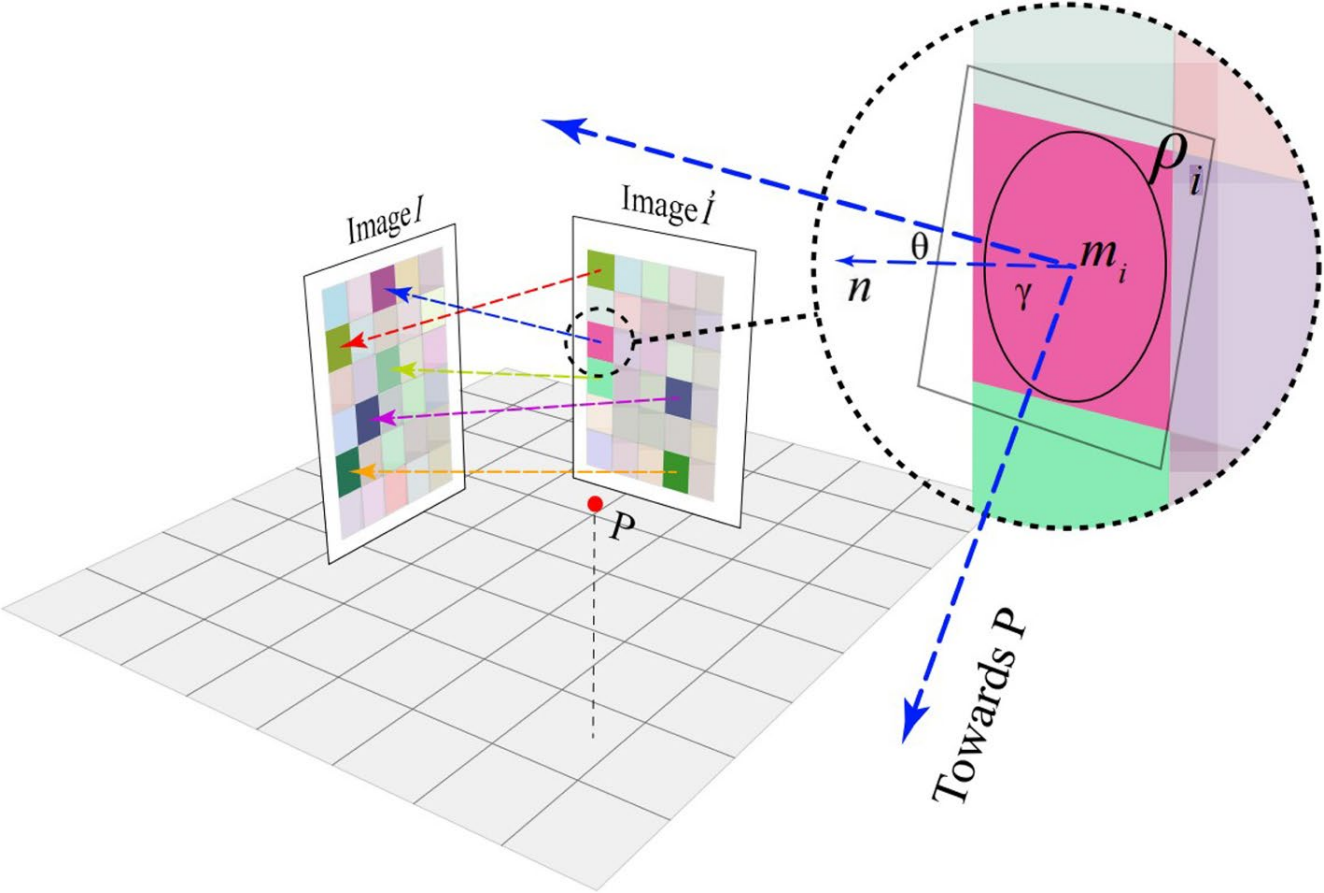
The device made by Hosseini et al. [22]. consists of one image *I* served as a palette and a set of mirror arrays to display *I*^´^. When the user looks at the mirror at the viewpoint *P*, the mirror reflects colors from *I* to form *I*^´^

Instead of reflecting the light source, Hosseini et al. [22] decided to use mirror grids to decompose and reorganize the object. Specifically, their method requires two images: one target image and another used as a palette to offer RGB values (Fig. 11).

**Fig. 12:**
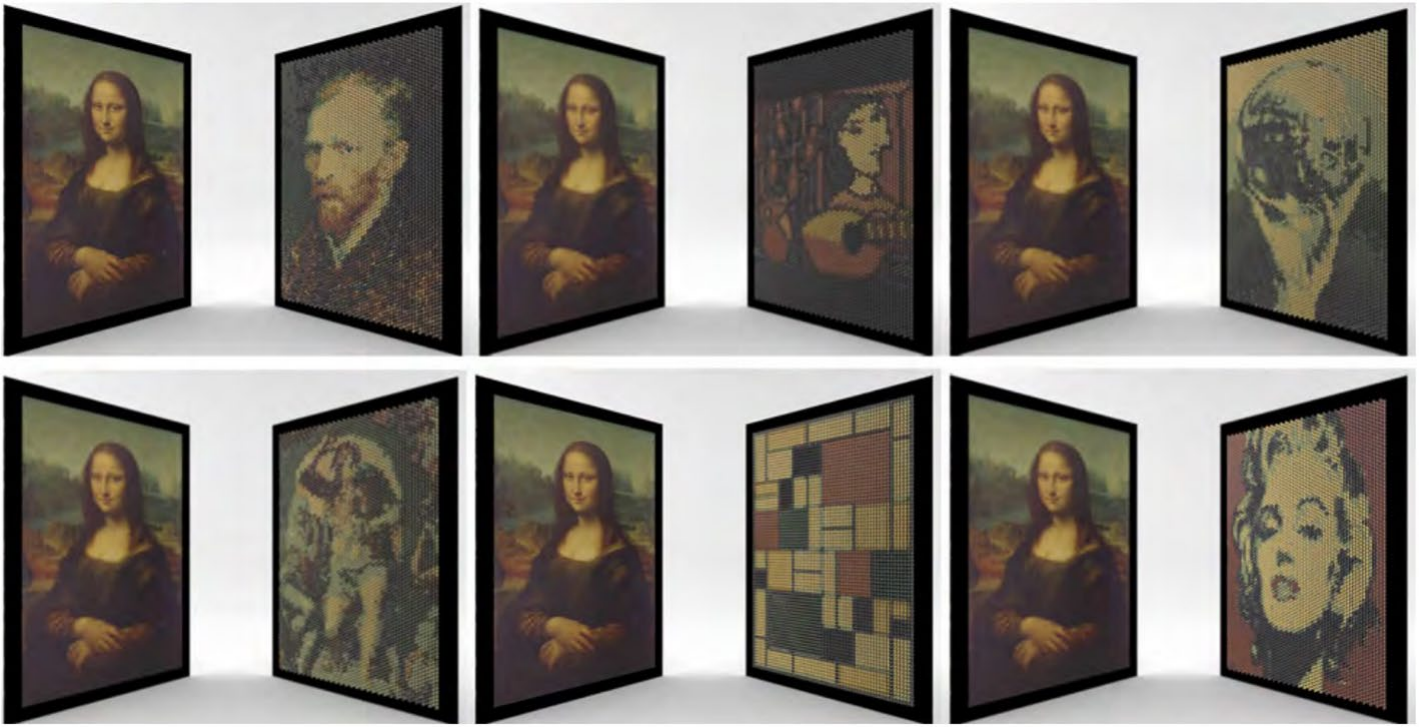
Six results generated by Hosseini et al. [22]. Their device uses a set of mirror arrays (right, in each panel) to reflect colors from the palette image (left, in each panel) to display the target image

Their algorithm first divided the input images into several cells. They calculated and stored the average pixel values of each block and its neighborhood for the palette image. This step aims to make the result more stable when there is any degree of deviation from the privileged point. Then, they applied a match-find algorithm to find ten blocks in the palette image whose average pixel value was closest to that of the blocks in the target image as the candidate blocks. Then, among these candidate blocks, they selected the block with the smallest variance of pixel values as the corresponding block. According to the correspondence between blocks, they could directly calculate the configuration of mirror grids. In their paper, they presented the assembly results of the device (Fig. 12). Their algorithm can reconstruct the target image well and consider the results’ stability when the viewpoint has offset. However, since they used a traversal search method to solve the problem, the resolution of the reconstructed images is low.

#### Internal reflection

**Fig. 13:**
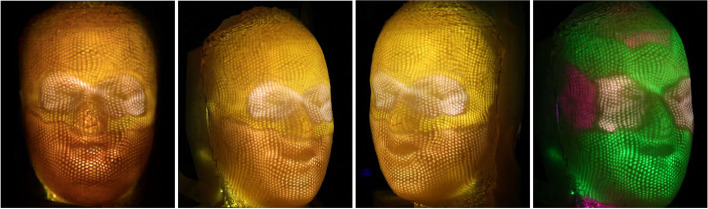
The result generated by Pereira et al. [22]. They used fibers to control the path of the light so that the light has a desired distribution inside the object

Pereira et al. [23] chose another way to utilize specular reflection. Their algorithm can control the light path inside a solid object using the total internal reflection (Fig. 13). To better model the problem, they tested different fiber configurations, including different materials and cross-section shapes, and also measured the transmission change with length and curvature.

The algorithm takes two parameterized surfaces as input and calculates the fiber paths between them. It minimizes fiber curvature to ensure a large enough light transmission. In addition, it also optimizes fibers’ distribution and constrains fibers from reaching surfaces vertically. Their work provides a new tool for the artist to create 3D visual optical art. However, they cannot guarantee that the fiber will always connect the input and output surfaces. In the future, using an explicit algorithm, which represents fibers as spline curves, may help solve this problem.

### Optimizing object

Reflected objects are another important part of reflection visual art. By modifying the shape or color of the object, the artist can show an unexpected object to the user. This strong contrast has made this class of artwork a widespread favorite.

Making this class of visual art is difficult for newbies. In the past, the artist would determine the shape and texture of the object by calculating the light path in advance on the manuscript. With the development of computer technology, people began to use algorithms to replace this process. The work of De Comite and Grisoni [24] is an initial exploration of this field. Their studied problem is simple. They only considered the reflected view without the constraints of other views. Therefore, their problem can be directly solved by using raycasting.

#### Multiple images

Wu et al. [25] researched the design problem of mirror cup and saucer art. Mirror cup and saucer art is a visual reflection art. By designing the shape and texture of the saucer, the artist can make viewers see an image directly on the saucer and another image on the mirror cup. However, producing mirror cup and saucer art with desired images and saucer surfaces is difficult since not all saucer shapes can adequately resolve the conflict between the reflected and directly viewed areas.

**Fig. 14:**
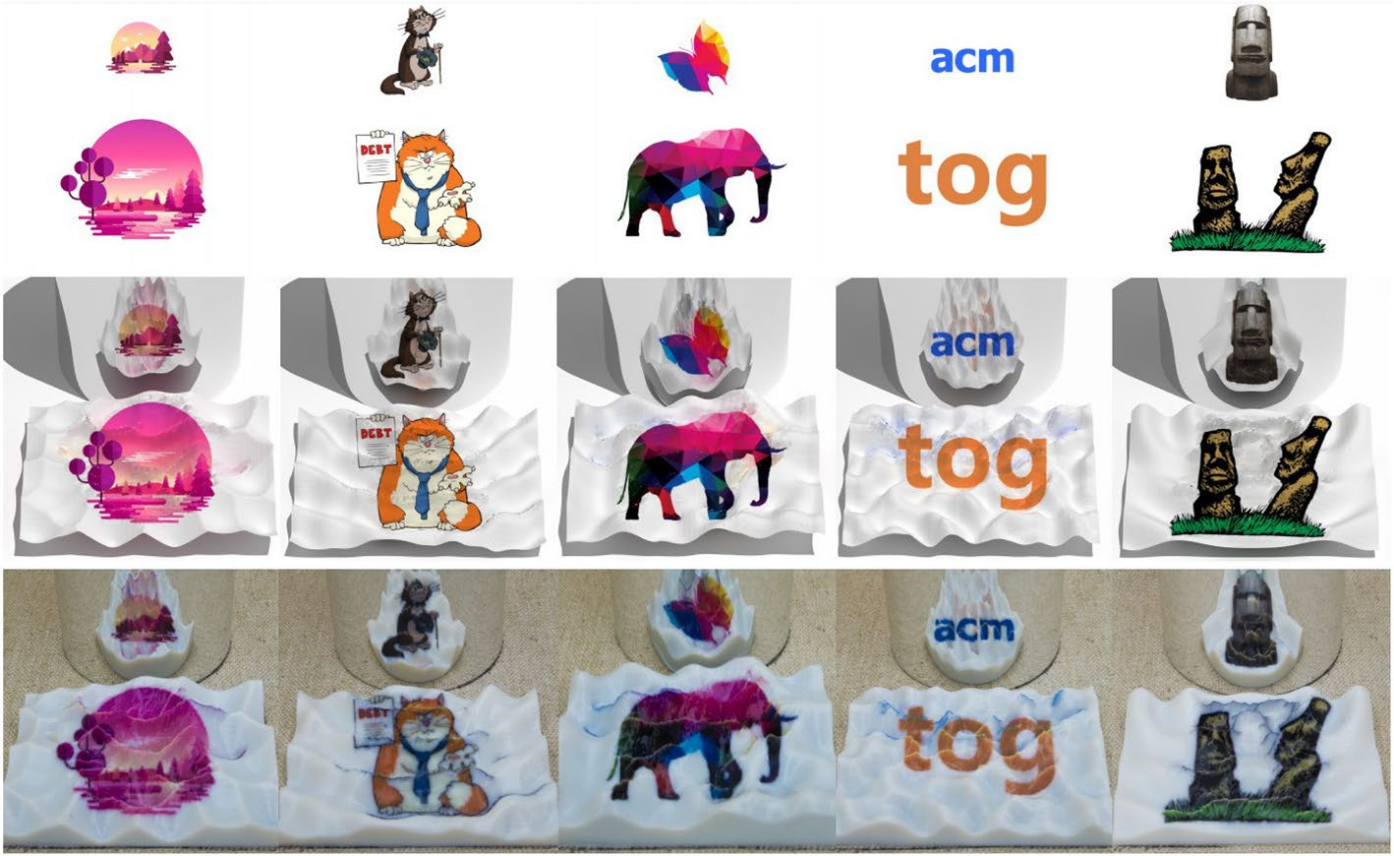
Five mirror cup art pieces physically fabricated with 3D printing in ref. [25]. Their algorithm deforms the surface appropriately to eliminate the conflict area. The user can see two different images when placing the result in front of a mirror cup

To address this issue, Wu et al. applied a two-stage optimization strategy. In the first stage, they used a Laplace operator energy to ensure that the surface shape would not be significantly deformed. In the second stage, sparse energy was chosen to ensure that as few vertices as possible were moved to reduce the image error further. They also introduced a black-white enhancement technique to reduce the conflict areas. In the optimization process, in order to obtain the derivation from images to the saucer, they used a differentiable rendering algorithm, Soft Rasterizer (SoftRas) [26], to render images. The reflected shape of the saucer was calculated in advance to avoid the disadvantage of SoftRas, which could not render the reflected images. They validated the produced art pieces by fabricating the colored saucers using 3D printing (Fig. 14). Their algorithm’s results are satisfactory, but there are also some limitations. First, they have no constraints on viewpoint stability, which leads to large distortions in the results when the viewpoint has offset. Second, they also do not consider the texture’s continuity, which makes the plate less pleasing when viewed from other viewpoints.

The problem of Sakurai et al. [27] was similar to that of Wu et al. Their results showed different images when viewed from different directions. To achieve this goal, they generated microstructures on a reflector. These microstructures consisted of colors and walls.

Their method first subdivided the surface of the reflector into grid cells with the same resolution. Then, walls were created to subdivide these cells into subcells so that the subcells only can be seen when viewed from the corresponding directions. Their algorithm had two steps. In the first step, they determined the location and height of each wall by solving an optimization problem. Their objective function consisted of three terms. The first term reflects the light as strongly as possible for better visibility, the second is to reduce the ghosting effects, and the third is to display each target image with as similarly reflected radiances as possible.

**Fig. 15:**
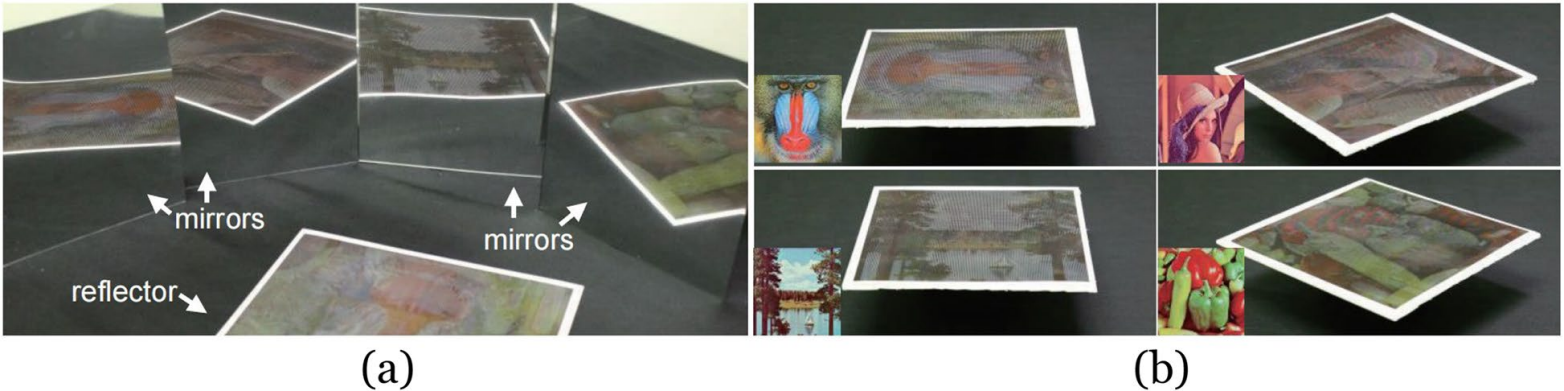
**a** The user can see different images when viewing the object generated by Sakurai et al. [27] through mirrors. **b** The result of viewing the object at different viewpoints

Due to the lack of gradient, genetic algorithms [28] were used to solve this optimization problem. After the geometric structures of cells were obtained, the colors of cells were calculated by optimization to better match the target images. Compared to the previous methods, the method proposed by Sakurai et al. does not require special hardware and/or materials to fabricate the reflectors. A standard ultraviolet printer (UV printer) is sufficient for the fabricating task (Fig. 15). Their algorithm fixes the height of the walls for simplicity. If we want a higher-contrast result, we can use a different configuration of walls for each pixel.

#### Multiple shapes

**Fig. 16:**
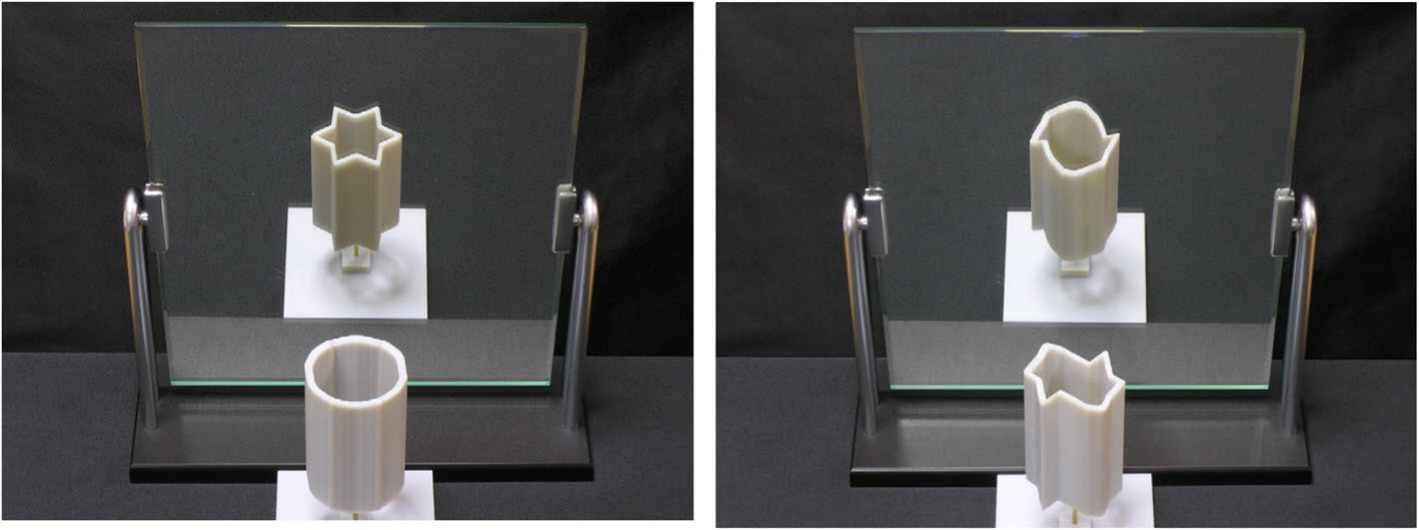
By properly placing the object in front of the mirror, the user can see a totally different object in the mirror [29]

Visual deception is also one of the techniques commonly used in reflection visual art. By carefully designing the object’s shape, the artist enables the user to see completely different shapes from different directions (Fig. 16).

Sugihara studied this phenomenon in a series of his works and proposed methods for generating artwork with visual deception [29–32]. Their design is based on two observations. One is that there are countless 3D curves corresponding to a certain 2D projection. Another is that humans tend to interpret 3D objects as having vertical and parallel geometric structures. He devised an algorithm to generate such impossible objects from the above two observations. It can be obtained by intersecting the visual hull of two target images. However, his algorithm’s limitation is that the result may not exist for some input images.

### Optimizing reflectance

There is also a class of visual reflection art that does not need mirrors. This artwork is an object whose reflectance is modified to appear special under light sources. By adjusting the object’s reflectance, the artist can reveal different patterns under different light sources and make the pattern be seen as a real 3D object.

#### Drawing board

Sugiura et al. chose to use carpets [33] or grass fields [34] to generate visual art. Their works are based on an observation: the reflectance of the fur varies with the height of the fiber lift. Their technology turns the blanket or grass in our lives into rewritable displays. Anyone can easily draw desired patterns on these objects using the devices they generate without worrying about damaging them.

**Fig. 17:**
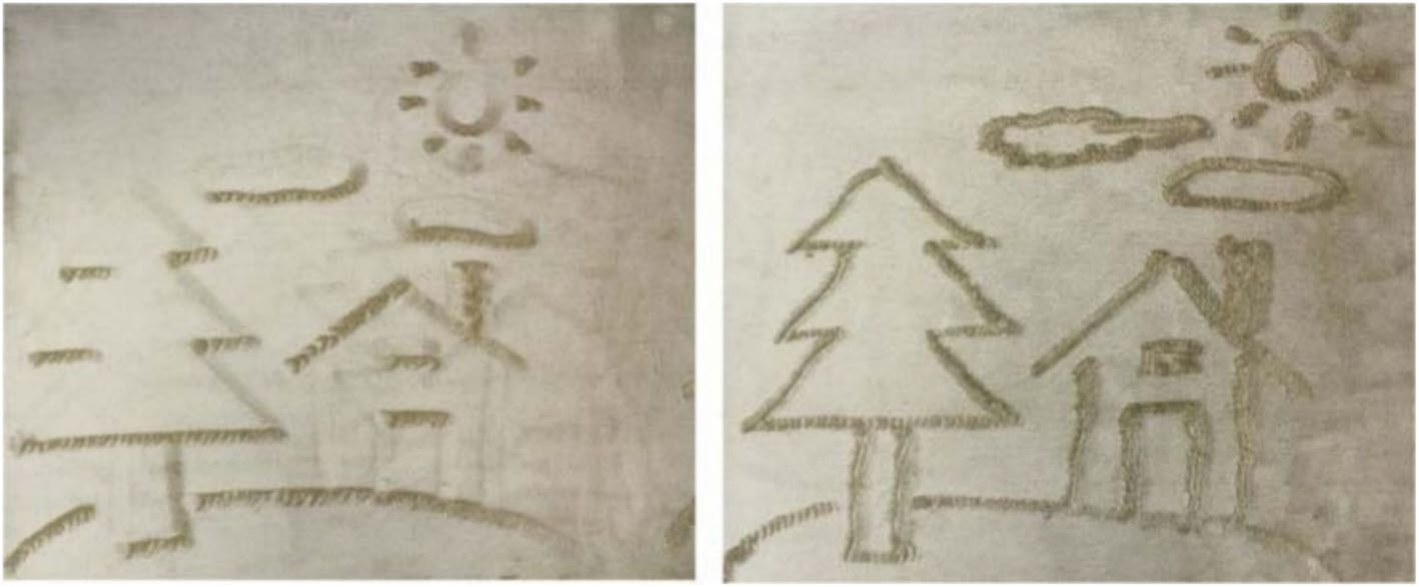
Patterns drawn on the blanket with fingers are blurrier than that drawn with the device [33]

They developed three different devices to draw patterns by utilizing these material properties. Compared to using fingers or other objects, using their devices to draw patterns is more convenient, and the drawn pattern is clearer (Fig. 17).

#### Bas-relief

**Fig. 18:**
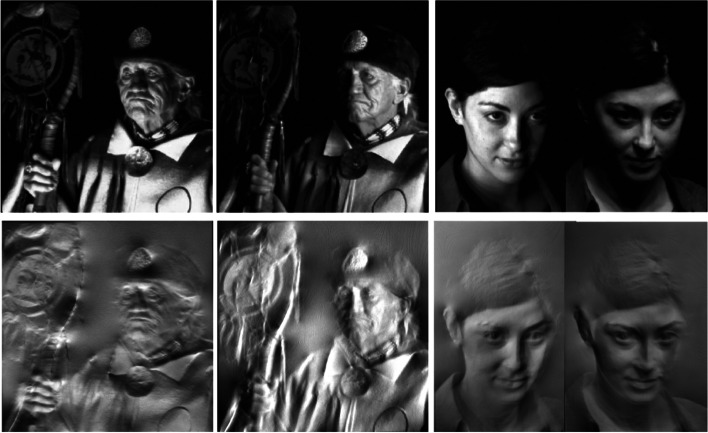
The bas-reliefs fabricated by Alexa and Matusik [35] have multiple appearances under different light conditions

Alexa and Matusik [35], Chao and Aliaga [36], and Zhang et al. [37] researched the generation of bas-relief under specific light sources. The algorithm proposed by Zhang et al. [37] considers the effect of lighting on the appearance of bas-reliefs. The bas-reliefs they generate are closer to the target image under the specified illumination condition. The works of Alexa and Matusik [35] and Chao and Aliaga [36] studied how to hide a second image in bas-relief. The hiding image can only be displayed under a certain light source. They both used a triangular mesh to represent the bas-relief heightfield. By optimizing the height of the heightfield vertices, they could control the angle between the normal of each triangular face and the light ray, thereby changing the brightness of each triangular face under light sources. The algorithm of Chao and Aliaga [36] has two energy terms. One is appearance loss, and the other is smoothness term. The energy function of Alexa and Matusik [35] is more complicated. It consists of five terms. Terms 1 and 2 are image gradient errors. Terms 3 and 4 are the first and second-order smoothness terms, respectively. Term 5 is used to lower large height values. Therefore, their reconstruction results are more refined compared to those of Chao and Aliaga [36]. They produced some results to demonstrate the effectiveness of their algorithm (Fig. 18).

#### Anisotropy

The works of Malzbender et al. [38] and Snelgrove et al. [39] studied how to design the reflectance of an object so that the pattern on its surface can be seen as a real 3D object under different light conditions. They both generated microstructures on the object to modify reflectance.

**Fig. 19:**
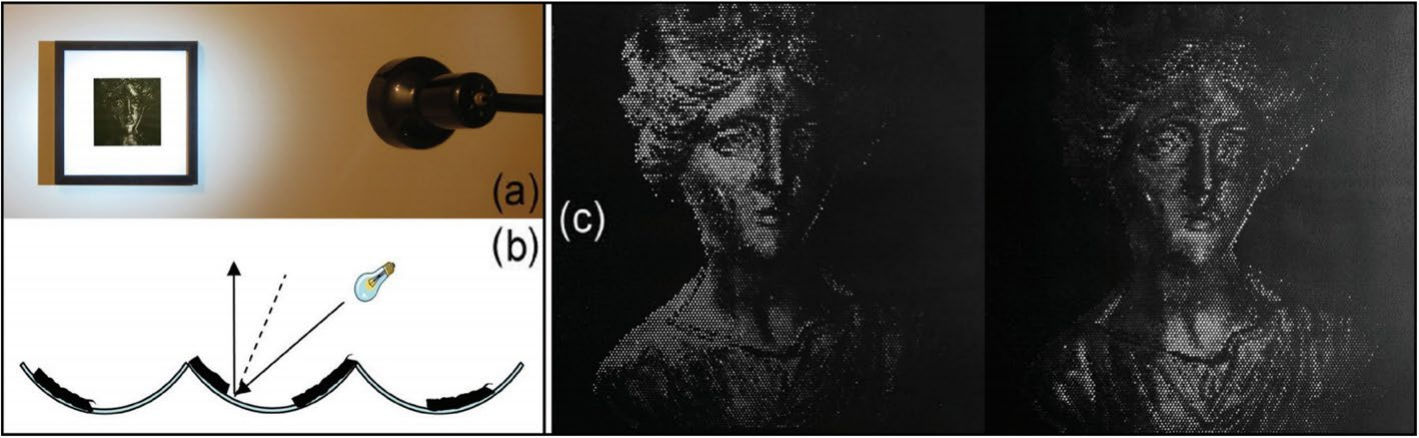
Malzbender et al.[38] presented an algorithm for producing paintings whose reflectance is similar to a real 3D object

Malzbender et al. [38] used a hexagonal array of spherical depressions to form the surface (Fig. 19). Each element has a specularly reflective surface. By selectively printing opaque or partially opaque ink on this surface, they could control the direction and amount of lighting under which a specular highlight would return. Their method can produce a spatially varied reflectance for an image. However, their results lack color, and the highlights of the results are very noticeable due to the use of specular reflections.

Snelgrove et al. [39] chose to create ‘walls’ to modify reflectance. When the incident direction of the light is nearly parallel to the wall, more light will be reflected, and the image will be brighter; otherwise, the image will be darker. They used this easy-to-fabricate structure to change the object’s appearance under different light sources. Compared to the work of Malzbender et al. [38], their results are color images and do not have highlights. Besides, they can alter the geometry of the objects in images and preserve the colors or alter the colors and preserve the geometry. However, in most cases, there are still some artifacts in the output images as most content is not in the achievable gamut.

Lan et al. [40] controlled reflectance by optimizing the surface heightfield. Their algorithm can generate surfaces with desired spatially-varying reflectance. By modifying the orientations of the facet and printing different ink to facets, they could reconstruct a wider range of reflectance than the printer gamut, including anisotropic materials. For manufacturing requirements, they added constraints on both geometry and ink gamut. They fabricated the resulting surface with commercially available hardware, a 3D printer to fabricate the facets, and a flatbed UV printer to coat them with inks. Compared to the two works above, their results have higher contrast and color saturation because they create anisotropic reflectance by modifying the surface normal. However, they cannot modify the object’s reflectance as drastically as the above two works.

## Refraction

The refraction of light is a phenomenon in which light is deflected when passing obliquely through the interface between one medium and another or a medium of varying density. Utilizing this phenomenon, the artist can control the light path to make a caustic image or deform an object. However, unfortunately, it is not easy to design a refractor that meets the requirements. Humans cannot observe the refraction phenomenon directly, leading to the artist having to revise their artworks through trial and error. The challenges in designing refraction visual art have attracted the attention of researchers.

### Caustics

**Fig. 20:**
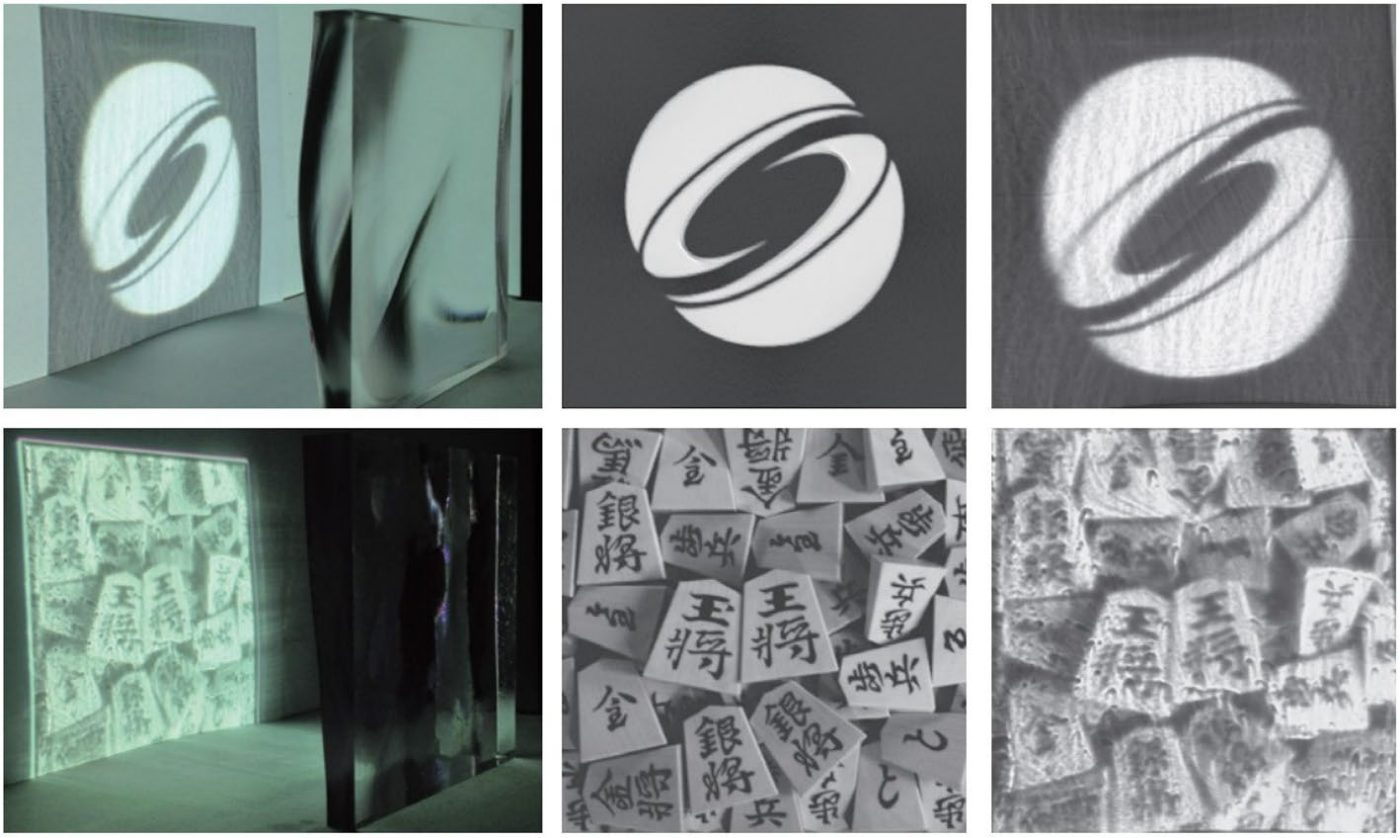
Yue et al.[41] used a prism to refract light into the desired pattern. From left to right: the result of their method, the target images, and the caustic patterns

Researchers mainly focus on caustics, where the artist uses one or more designed lenses to refract the light to form a caustic pattern on a receiver (Fig. 20). This is a typical inverse problem. The difficulty in solving this problem is that we cannot obtain the gradient from images to the lens shape, making it hard to optimize the lens shape using the error function.

Finckh et al. [42] researched this design problem in 2010. Generally, the simulated annealing (SAN) algorithm [43, 44] is used to solve this problem. However, they found that the simultaneous perturbation stochastic approximation (SPSA) optimization algorithm [45] is more robust and efficient. They also used the GPU to accelerate caustic rendering. Their algorithm can cope with various surface representations, but it is not well integrated with the rendering framework, and the renderer is only used as a tool for calculating errors. In addition, the SPSA and SAN may fail to solve their problems.

The following year, Papas et al. [46] proposed their solution. They decomposed the input image into (possibly overlapping) anisotropic Gaussian kernels. This decomposition was then used to generate a set of continuous surfaces. Each surface focuses light onto its corresponding Gaussian kernel. Expectation maximization [47] was applied to optimize their problem. Their method can produce a near-continuous surface. Moreover, compared to the work of Finckh et al. [42], it is more stable and can handle more extreme cases.

Later, Kiser et al. [48, 49] studied this design problem. Their algorithm simplified the lens to a heightfield. The error between the caustic image and the target image was reduced by optimizing the normal of each facet. Once the normals were computed, they integrated them to get a heightfield as the output surface. Their algorithm uses a gradient-based solver and considers the effects of errors in the manufacturing process. Their results are better than the previous ones.

**Fig. 21:**
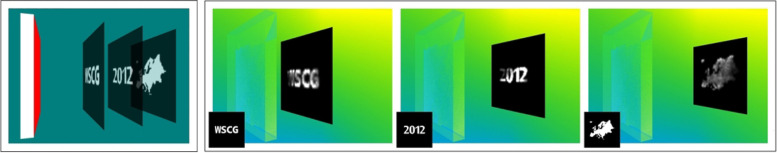
The approach proposed by Tandianus et al.[50] allows the user to create an object that can form multiple caustic patterns at different distances

The problem that Tandianus et al. [50] studied is more complicated. The objects produced by their approach can generate different caustic patterns at different distances (Fig. 21). To solve this problem, they subdivided the object and caustic patterns into regular cells. The light ray would pass through one caustic object cell and one cell of each caustic pattern. Their goal was to then calculate the combination of the refracted light to better approximate the input patterns. Once they had determined the combination, they could compute the normal of the surface and reconstruct the object. However, a good result for this problem may not exist. Therefore, they proposed a method that optimizes the size and position of patterns and expands patterns to improve the algorithm. This reduces image errors to some extent but does not guarantee a satisfactory result.

In 2014, Yue et al. [41] proposed a method to generate a continuous surface for the caustic. Their method involves two steps. First, they calculated a smooth and light-preserving energy mapping between the distributions of the light on the object and the light on the receiver. This problem can be regarded as a parameterization between two planes and solved using a differential geometry approach. Second, they utilized this mapping to compute the surface of the object. Since the mapping was continuous and smooth, they obtained a smooth refractive surface, different from previous algorithms. Their results were better than those of Finckh et al. [42] and Papas et al. [46].

Schwartzburg et al. [51] also presented their solution in the same year. They used an optimal transport map to handle the mapping problem. This map determined each light ray’s path. Using Snell’s law, they could compute the surface normals and then integrate them to produce the final result. Their method can generate high-contrast images, including completely black areas and point and curve singularities of infinite light density. These features have not been shown in any previous method.

Lancelle et al. [52] found that lens dispersion could be interpreted as stereoscopic disparity. When the light was refracted through a lens, the relative dispersion would appear between the cyan and red wavelengths. This phenomenon could be used to produce a 3D image when viewed through anaglyph glasses. Their algorithm used a prism array to create a caustic image, which optimized the shape and configuration of the prism array to make the caustic image closer to the target image. They were the first to use caustic images as 3D images.

Berry [53] limited his problem to the case where the ray deflections were sufficiently small. In that case, the caustics between the object and the receiver can be ignored. Thus, he could use the Laplacian function of the relief height to approximate the caustic image. Therefore, the desired relief could be obtained by solving Poisson’s equation. With this simplified model, the caustic problem can be solved easily. However, his results are not as good as those of the previous methods.

Meyron et al. [54] presented a generic and parameter-free algorithm to solve caustic design problems with reflection or refraction. They found that these problems are related to optimal transport, which can be seen as solving the problem of light intensity distribution while maintaining light energy conservation. These problems can be handled by computing visibility diagrams whose structures are the same. To get these diagrams, they intersected a 3D Power diagram with a planar or spherical domain. This allowed their algorithm to solve these kinds of problems efficiently.

Suzuki et al. [55] presented a method for controlling the surface of liquids to generate caustics. The motivation of their work is to study how to compute the initial force that deforms the surface of the liquid to a target shape. As the liquid surface should be continuous, they chose to use the Poissons equation to solve their problem. Finally, their algorithm was confirmed by fluid simulation.

With the rise of differentiable rendering in recent years, researchers have new tools to solve the caustics problem. The versatile renderer, Mitsuba 2, proposed by Nimier-David et al. [56], can be used to address this issue. Since this renderer supports differentiable rendering, the gradient descent algorithm can be applied to solve the design problem.

Later, Kassubeck et al. [57] presented an algorithm using the differentiable ray-tracing technique. However, they found that purely using differentiable rendering to solve the caustics problem is inefficient and not robust enough. Therefore, their follow-up work developed a learning-based extension [58]. They added two learned components to their algorithm. The first is a denoiser used to fix the number of forward simulation samples during Monte-Carlo integration to lower the runtime cost. The second is a learned gradient descent scheme that can reduce many steps while keeping the shape error within an acceptable range.

### Volumetric displays

**Fig. 22:**
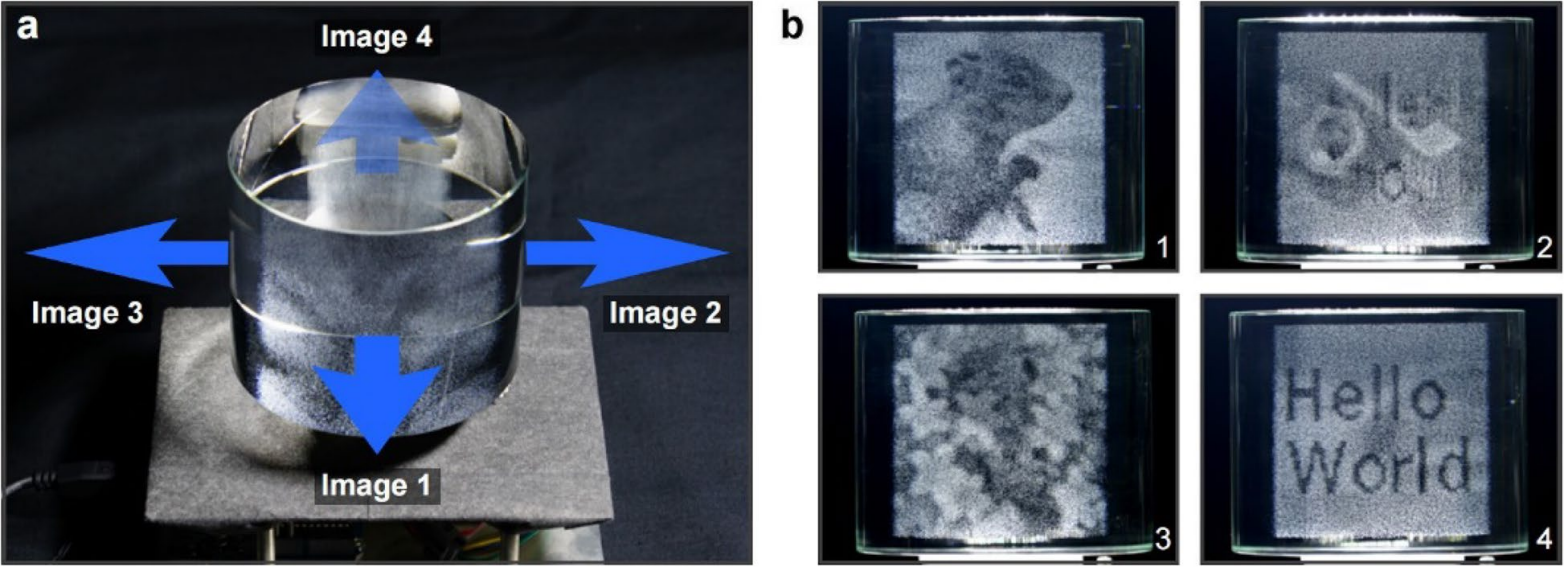
**a** Volumetric displays can project different images in different directions [59]; **b** Four images displayed by a cylindrical 3D crystal

Volumetric displays can directly render a 3D image in space (Fig. 22). This novel display gives artists a higher degree of freedom to present images. Similar to the Shadow art [2] we mentioned before, the artist can display multiple images in the same space using a volumetric display. However, the volumetric display may some- times be made of transparent materials such as glass. The influence of refraction needs to be considered when designing the display.

Nakayama et al. [60] continued to study this problem for several years. In the beginning, they designed the volume display as a polyhedron, ensuring that each viewing direction was orthogonal to the display’s surface, and then ignored the effects of refraction. For each voxel color value $V$ with $N$ input images, it was defined by:

1 $$V=\lambda p\left(1\right)p\left(2\right)p\left(3\right)p\left(4\right)\cdots p\left(N\right)$$

where $\lambda$ is a constant used to adjust the total intensity and $p\left(1\right)$, $p\left(2\right)$, $p\left(3\right)$, $p\left(4\right)$, $\cdots$, $p\left(N\right)$ represent the pixel values of the points at which lines from the voxel center perpendicularly intersect the original patterns. The pixel value in the projected image from the volume was then denoted as the sum of its corresponding voxel color values. Then, they calculated $\lambda$ to minimize the image error.

However, the image quality of the patterns reconstructed by this algorithm was limited. Later, they proposed an iterative algorithm to improve it [61]. Their algorithm was as follows:

2 $$V{\left(x,y,z\right)}^{\left(k\right)}=\prod_{i=1}^{N}\sqrt[n]{{I}_{i}{\left({u}_{i},{v}_{i}\right)}^{\left(k\right)}}$$

3 $${P}_{i}{\left({u}_{i},{v}_{i}\right)}^{\left(k\right)}=\sum_{{w}_{i}}V{\left(x,y,z\right)}^{\left(k\right)}$$

4 $${I}_{i}{\left({u}_{i},{v}_{i}\right)}^{\left(k+1\right)}={I}_{i}{\left({u}_{i},{v}_{i}\right)}^{\left(k\right)}\frac{{I}_{i}{\left({u}_{i},{v}_{i}\right)}^{\left(0\right)}}{{P}_{i}{\left({u}_{i},{v}_{i}\right)}^{\left(k\right)}}$$

where $N$ is the number of the input images, $k$ represents the iteration number, ${P}_{i}{\left({u}_{i},{v}_{i}\right)}^{\left(k\right)}$ is the pixel value of the output pattern in ${k}^{\mathrm{th}}$ iteration, ${I}_{i}{\left({u}_{i},{v}_{i}\right)}^{\left(0\right)}$ is the ideal pixel value of the input pattern, $\left({u}_{i},{v}_{i}\right)$ is the coordinate of the corresponding projection plane of the voxel $\left(x,y,z\right)$, and ${w}_{i}$ is the exhibition direction of the $i$ th exhibited pattern. Compared with the previous algorithm, the image quality of the patterns is improved by iterative computation.

In a follow-up, they presented a method to construct a full-color volumetric display using a commercially available inkjet printer [62] but did not improve existing algorithms. Next, they proposed an algorithm to generate a volumetric display using strings [63]. Their device uses a projector to project light onto an array of strings so that multiple images can be viewed in different directions. The occlusion relationship between the strings needs to be considered when dealing with this problem. They achieved the arrangement of strings using a computer simulation under constraints.

In 2019, they presented a new refraction-based approach [59] to embed multiple images into a single volume structure rendered on a glass solid (3D crystal). The main improvement is that refraction is taken into account when calculating the projected coordinates of the voxel, and two constants, ${C}_{1}$ and ${C}_{2}$, are added to the numerator and denominator of Eq. (4), respectively, to prevent division by zero. However, this algorithm has a problem. Once the input images contain a pure black image (all the pixel values are ‘0’), the voxel color will always be black. To address this issue, a revised algorithm [64] that could display such images was presented. The following addition-based equation was adopted:

5 $$V\left(x,y,z\right)=\sum_{i=1}^{N}{I}_{i}\left({u}_{i},{v}_{i}\right)$$

Therefore, the iteration formula became an iterative addition–subtraction approximation:

6 $${I}_{i}{\left({u}_{i},{v}_{i}\right)}^{\left(k+1\right)}={I}_{i}{\left({u}_{i},{v}_{i}\right)}^{\left(k\right)}+\left({I}_{i}{\left({u}_{i},{v}_{i}\right)}^{\left(0\right)}-{P}_{i}{\left({u}_{i},{v}_{i}\right)}^{\left(k\right)}\right)$$

They succeeded in fabricating a directional volumetric display capable of displaying separate images from different directions when a pure black image is included.

### Other refraction art

Here, we cover the rest of the work of refraction visual arts. They are mainly divided into two classes. The first involves utilizing refraction to show a new pattern by deforming the original one. The other involves deflecting light to create the desired pattern on the receiver.

#### Multiple images

Papas et al. [65] presented an approach to generate devices that can deform the original pattern to display hidden images. Their devices consist of a lens array. It may show multiple hidden images when placed on the source image in different orientations. Their algorithm also supports generating the universal lens, used with arbitrary source images.

**Fig. 23:**
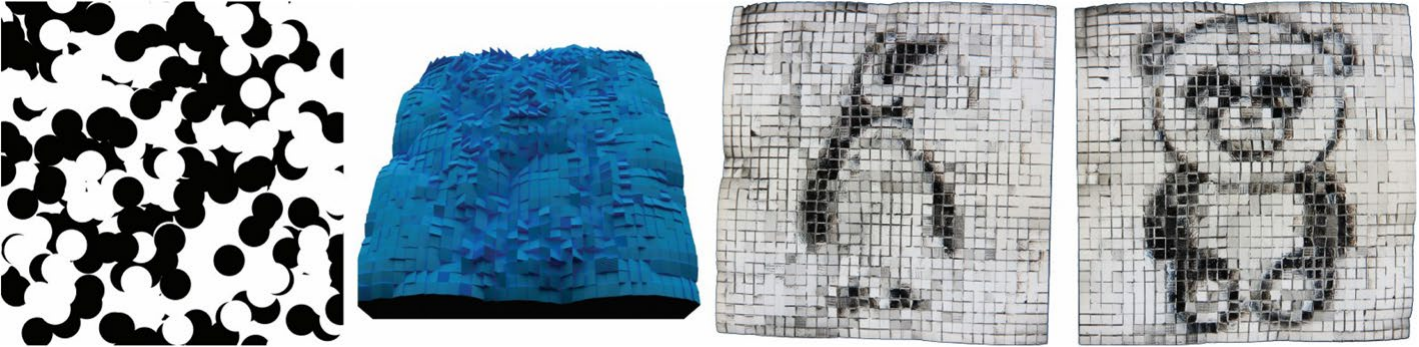
From left to right: source image, lens, and the result images when the lens is placed over the source image in different orientations [65]

The first step of the algorithm is to assign each lens facet to a unique source image patch. An image color matching term and a smoothing term used to avoid steep normals were used to find suitable facet-to-patch matches. Search space was restricted to a preset neighborhood. In the next step, they assigned an orientation to each facet. In the third step, they used SAN to refine the previously generated results and then performed the fourth step to optimize the overall height to ensure the smoothness of the surface. They validated their simulation results with many real-world manufactured magic lenses (Fig. 23). However, their method does not support full-color images as inputs. Moreover, the manufacturing error is optimized.

Zeng et al.[66] also utilized refraction to show several patterns from different angles. However, their research focused on manufacturing. Their system uses as input the 3D model and the user desired appearances at different viewpoints. Then, it optimizes the lens placements and underlying color pattern to display different appearances at different viewpoints. Their method tested the effect of lenses of different sizes and various post-processing techniques for the best fabrication configuration. They also made an interactive system to assist the user in designing their desired objects.

#### Puzzle

**Fig. 24:**
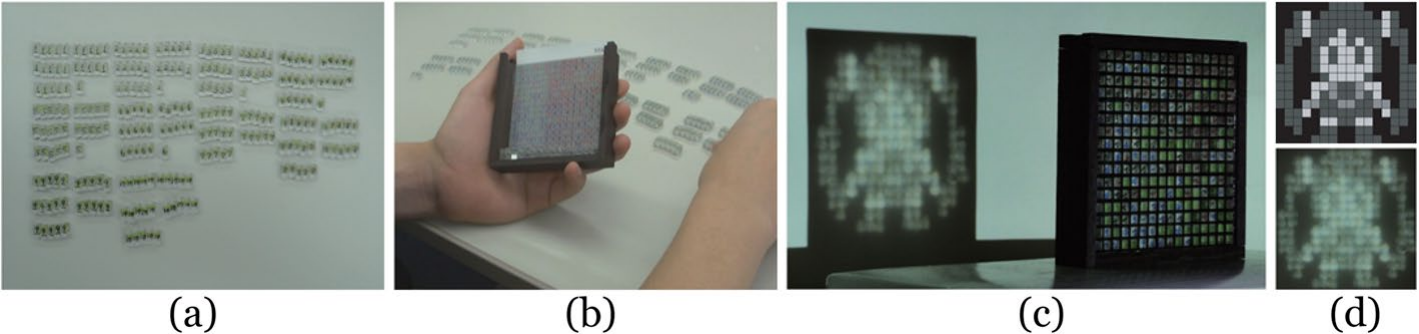
**a** Yue et al.[67] pre-prepared a fixed number and variety of sticks for the user; **b** Based on the input image, their algorithm automatically generates the arrangement of the sticks, and the user needs to manually assemble the sticks according to the results; **c** Once assembled, the device refracts light into the input image; **d** Top: the input image; bottom: the image on the screen

Yue et al. [67] proposed a method for generating a puzzle type of refraction visual art (Fig. 24). Their device consists of sticks made of acrylate resin. When the light from a parallel light source passes through them, it will be refracted in certain directions. From an entertainment point of view, they made the number of sticks the user had in advance equal to the number of pixels in the target pattern. Once a user inputs an image, their system will automatically compute a combination of sticks to refract the light for displaying this target pattern on the receiver. The quadratic norm of the image error and its gradient were used in the objective function. Then, based on the result, the user could assemble the sticks to enjoy creating visual art. However, when the intensity of the input image is far different from that of the incident light, their algorithm may fail.

#### Dispersion

**Fig. 25:**
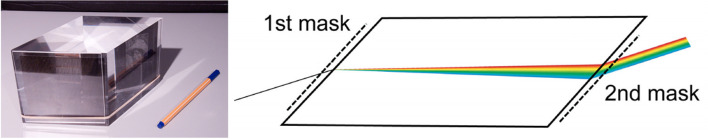
The device of Hostettler et al. [68] consists of a prism and two masks. The prism refracts white lights into colored lights, and masks control the intensity of the lights

**Fig. 26:**
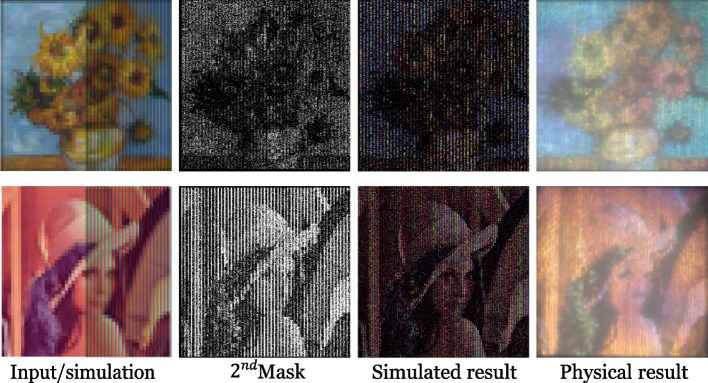
Two experiments of Hostettler et al. [68]. From left to right are comparisons of the input image and the simulated result, the second mask, the simulated result, and the fabricated result

Hostettler et al. [68] used the dispersion phenomenon to convert white light into a color image. First, they calculated the extent *e* of the dispersion of visible human light. Then, they set the width and length of each pixel to 3*/*2*e* so that every 1*/*3-pixel width light was refracted into a pixel-wide rainbow. Two transparent masks were used to control the color value of each pixel (Fig. 25). The first mask was placed at the incident surface of the prism(s) to control the brightness of small rainbows. The second mask was placed at the light- exiting surface of the prism(s) to control the intensity of light received per 1*/*3 pixel. The Levenberg–Marquardt optimization algorithm was then used to find a suitable configuration of these two masks to minimize the image error. They demonstrated their approach on a wide variety of images (Fig. 26). However, their actual results were not ideal due to the limitations of manufacturing accuracy.

## Others

Here, we introduce the remaining 3D visual optical artworks. These works of art take advantage of people’s misunderstanding of the depth of objects under specific light sources to create attractive artistic effects. The design problem of this type of artwork is also a reconstruction problem. Given the fixed light condition, we need to calculate the geometry and texture of the object to reduce the image error between targets and rendered images. However, compared to the standard reconstruction task, the geometry of this type of artwork is ‘wrong.’ We begin by reviewing works that generate anamorphic objects. Anamorphic objects can only be viewed correctly from a certain viewpoint. These works use some existing rendering algorithms or industrial design software to simplify the design process of creating these objects.

Then, we will cover the works of visual art about illusion objects and impossible objects. These works exploit prior human knowledge of 3D objects, causing users to come up with the wrong topology when faced with the wrong depth.

### Anamorphic objects

**Fig. 27:**
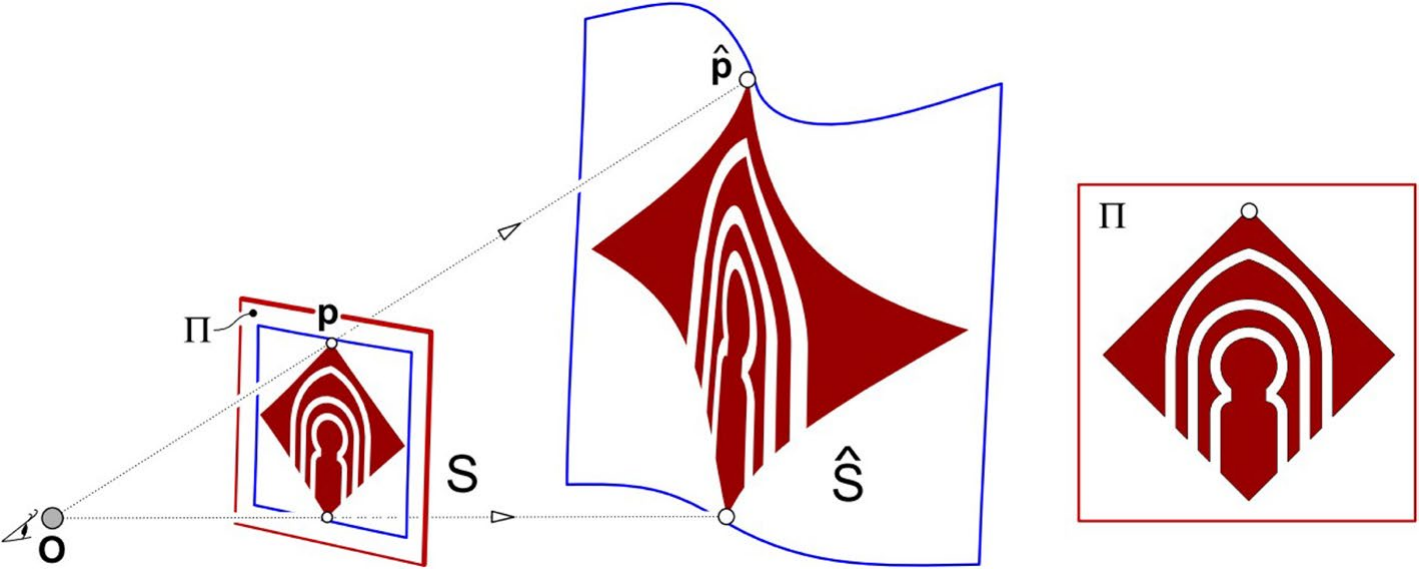
Sánchez-Reyes and Chacón [69] used rational B´ezier surfaces or volumes to create anamorphosis

In 2016, Sánchez-Reyes and Chacón [69] proposed the use of rational B´ezier surfaces or volumes as deformation tools to create anamorphosis, a kind of ambiguous object (Fig. 27). Their algorithm guarantees that the projection of the object from a user-specified viewing angle would not change. Using this algorithm, the user can easily and intuitively modify the object shape while satisfying constraints.

Symeonidou [70] reviewed the experiments and methods for constructing anamorphosis using computer aided design. They also introduced an educational approach that applies anamorphosis to teach students digital representation methods.

The following year, Hosseini et al. [71] introduced an algorithm to create anamorphic architecture. Their algorithm builds objects that satisfy constraints by reversing linear perspectival projection to convert the input 2D image into 3D space.

The above three works only make constraints on the projected image of the object under a certain viewing angle; therefore, it is not difficult to solve them.

### Illusion objects

An optical illusion is something that plays tricks on your vision. This kind of 3D artwork utilizes human perception of the 3D space and experience of the structure of 3D objects to mislead the user, causing them to draw wrong or even contradictory conclusions about the shape of the object.

**Fig. 28:**
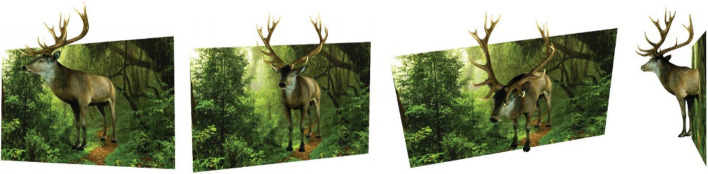
The sculpture fabricated by Arpa et al.[72] is a mix of 2D images and 3D objects

Arpa et al. [72] presented a method for generating objects consisting of 2D image and 3D components (Fig. 28). Given a 3D scene, they converted the user-specified part into 2D, while others remained 3D. The main challenge in this work was to make the transition between 2D and 3D parts smooth.

In their algorithm, the user first selected a cut plane, and the part of the scene between the cut plane and the rendering plane, called the region of interest (ROI), would remain 3D. Then, they deformed the boundary vertices of the 3D part onto the cut plane. Then, they created a grid to represent the rendering plane and attached the ROI to it. They used the 3D depth information of the 2D part and optimized the height of ROI to get a smoother transition between the 2D and 3D parts. Finally, they attached a texture to the resulting mesh. Their results preserved part of the 3D scene, allowing the user to get a more realistic 3D experience when viewing them.

**Fig. 29:**
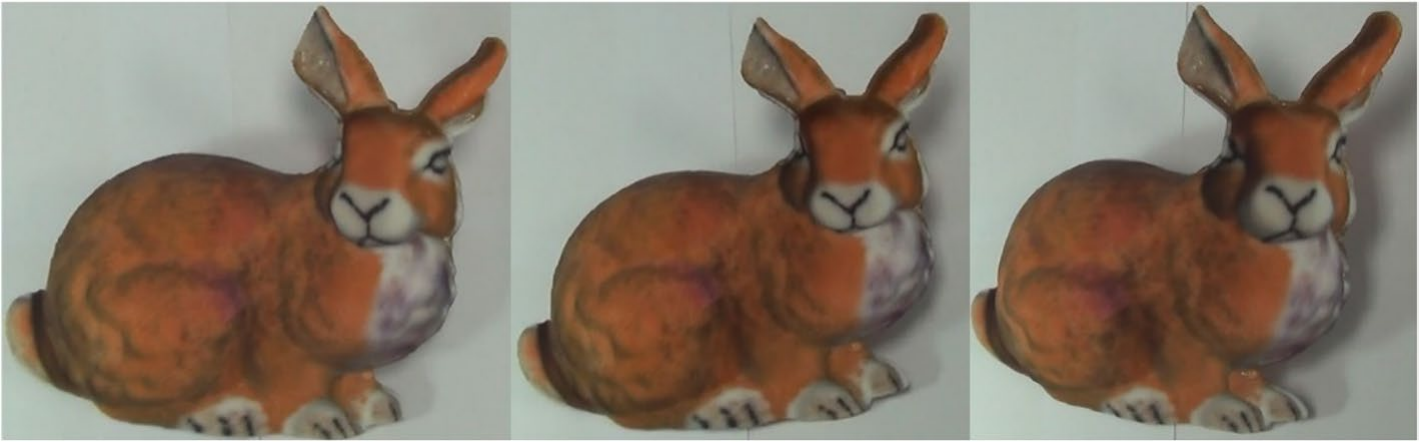
The bunny model was created using hollow-face illusion [73]. When viewing the object from different viewpoints, the user feels that the bunny’s head is turning

Tong et al. [73] presented a novel approach for creating self-moving objects using hollow-face illusion (Fig. 29). The key to this method is to take advantage of the fact that humans prefer to interpret faces as convex rather than concave. When we project a face texture onto a concave object and view it from different viewpoints, the “wrong” transformation we see is interpreted by our brain as the object itself moving.

Their algorithm employed an energy function composed of two energy terms to optimize the mesh shape. Term 1 is used to control the relative motion illusion of different parts of the concave surface and mimic the deformation of the convex one. Term 2 is used to preserve the concave surface to be a shape of a human face and to strengthen the hollow-face illusion. These energy terms represent ‘bottom-up’ visual signals and ‘top-down’ knowledge. Their work provides a qualitative and quantitative analysis of hollow-face illusion, which helps the artist create their hollow-face illusion works.

### Impossible objects

**Fig. 30:**
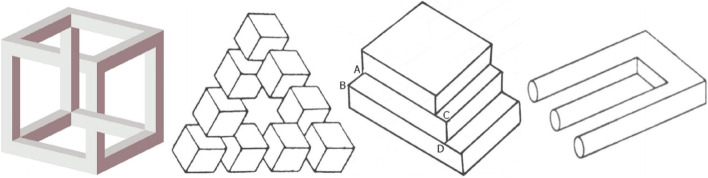
Different types of impossible objects in ref. [74]

The impossible object is also a kind of visual art. The artist mainly uses the depth misperception caused by projection to create topological structures that seem impossible to exist (Fig. 30). The modeling and rendering of such objects were studied by Wu et al. [74]. They believe that the optical illusion caused by impossible objects is due to the different projection transformations used in different parts of the object when generating the 2D image, while humans tend to interpret the image as using the same rigid projection transformation.

In their algorithm, the user first selected one part as the reference part and chose a desired viewpoint for the camera. The selected reference part was then rendered, and the other parts would be deformed by a non-rigid transformation to connect this 3D model. They used thin-plate spline (TPS) warping [75] to deform the object. They chose TPS because it minimizes the Laplacian (or curvature) of the warping energy, so the deformation is smooth and natural. They finally designed an interactive system to help the user model and render this type of impossible object. Elber [76] also worked on modeling impossible objects in 2011. In this work, they studied the principles of impossible objects and found a class of impossible objects that can be fabricated. They also presented a mini-modeling package for the user to create impossible objects. Their algorithm takes as input a normal 3D model and deforms it to produce an impossible object. Using his framework, users can easily model some simple impossible objects.

**Fig. 31:**
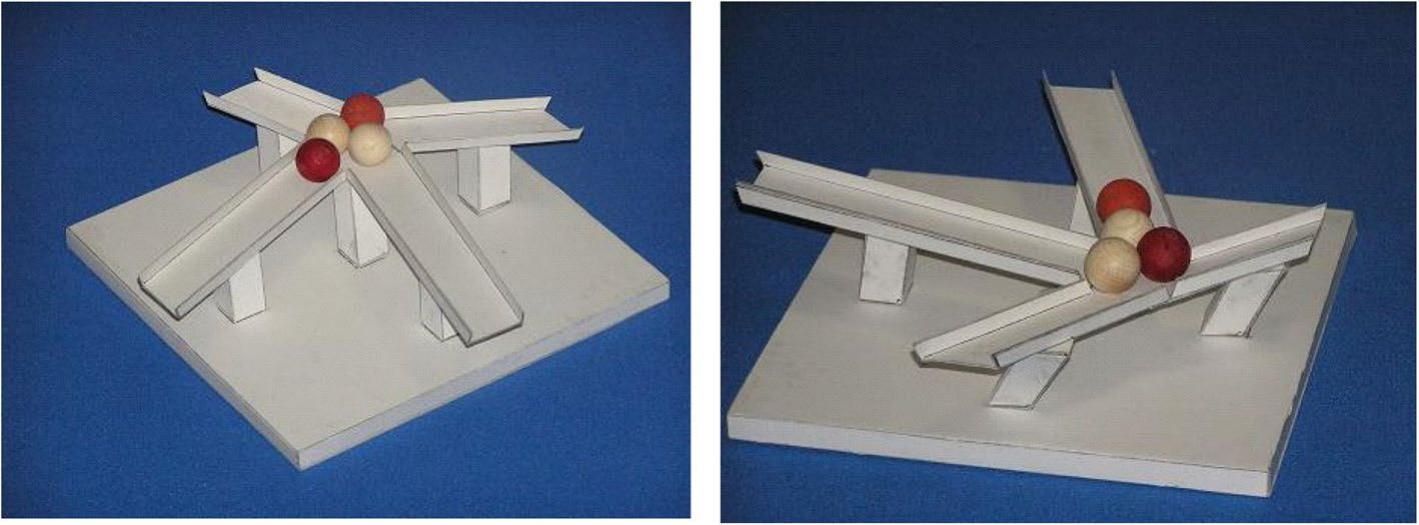
The “impossible motion” object created by Sugihara [77]

Sugihara [77] discussed how to design impossible objects. He presented a method for creating “impossible motion” objects. These objects contain slopes whose descending direction seems to be the opposite of the actual direction when viewed from a certain viewpoint. Therefore, when we put a ball on it, the movement of the ball appears to defy the laws of gravity (Fig. 31). This is possible because the 2D image lacks depth information and humans tend to interpret the shape of objects as familiar to them.

Sánchez-Reyes and Chacón [78] explored how to build these 3D objects with curved surfaces through the prism of computer aided geometric design (CAGD).

**Fig. 32:**
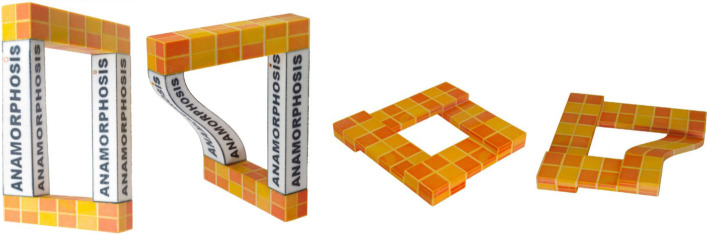
Two impossible objects of Sánchez-Reyes and Chacón [78] created using 3D printouts

They used standard CAGD techniques, such as homogeneous coordinates and non-uniform rational B-splines, to deform the object along radial lines through the viewpoint to create impossible objects. Using their algorithm, the user only needs to move the control points to deform the object. This interactive operation makes modeling impossible objects more intuitive. Moreover, their algorithm also guarantees texture consistency, which makes the results even more deceptive. They finally used 3D printing to confirm the reliability of the algorithm (Fig. 32).

It is not difficult to design this kind of artwork, so the research works are limited to how to model and render it more conveniently using computer technology.

## Results and discussion

Artists have a long history of making 3D visual optical art. Designing these artworks requires not only imagination and experience but also patience and a little luck. Generally, making a visual optical artwork is tedious and time-consuming, and artists need to go through trial and error to create a satisfactory result. Therefore, it is a valuable direction for researchers to propose algorithms to replace the manual trial and error process to create 3D visual optical art. These algorithms make it easier to create artworks and allow ordinary people to enjoy the fun of creating them [67].

Coincidentally, the connection between visual effects and three-dimensional objects in 3D visual optical arts falls within the research scope of computer graphics; therefore, these design problems are a natural fit for graphics researchers. They can all be treated as reconstruction and fabrication problems. Researchers need to calculate the correct geometry and texture of the object and fabricate it according to the input images. This section will review and summarize the previously introduced papers and analyze the problems they study. We also propose a general solution framework for these design problems at the end of the section. We hope our work will provide some inspiration for researchers interested in this field.

To facilitate researchers to continue their research in this field, we classify the 3D visual optical arts reviewed in this article according to their characteristics (Table 1). With a simple analysis of this table, we draw the following conclusions. First, researchers prefer to study the generation problem of ambiguous objects. We think this is because the manufacturing process for this type of artwork is complex. Therefore, it is more challenging and valuable to solve these design problems.

**Table 1:** Classification of the 3D visual optical arts reviewed in this paper

Art type	Reference	Ambiguous	Dynamic	Need receiver	Need decoding	Specific light source
**Shadow**						
Shadow art	[1, 9, 12, 14]			●		●
	[2, 4, 5, 10, 11]	●		●		●
	[6, 8]				●	
Self-shadowing images	[15]	●				●
	[16]					
**Reflection**						
Optimizing mirror	[17, 21]			●		●
	[22]	●			●	
	[23]					●
Optimizing object	[24]					
	[25, 27, 29–32]	●				
Optimizing reflectance	[33, 34]					
	[37]					●
	[35, 36]	●				●
	[38–40]	●				
**Refraction**						
Caustic	[41, 42, 46, 48, 49, 51–54, 56–58]			●		●
	[50]	●		●		●
	[55]		●	●		●
Volumetric displays	[59–64]	●				
Other refraction art	[65]	●			●	
	[66]	●				
	[67, 68]			●		●
**Others**						
Anamorphic objects	[69–71]	●				
Illusion objects	[72]					
	[73]	●	●			
Impossible objects	[74, 76, 78]	●				
	[77]		●			

Moreover, researchers prefer to propose automatic algorithms to create artworks rather than aid artists in designing artworks directly. Second, researchers pay little attention to generating dynamic 3D visual optical art. The artworks they generate are often static or need to be viewed from a fixed viewpoint. The production of dynamic visual optical art is quite difficult. However, we believe that graphics researchers can produce artworks that exceed artists’ abilities, as many computer graphics tools exist. Therefore, we think this may be a good direction for researchers in the future. Last, although designing decoding-related art is not a difficult task for graphics researchers, only a few works [6, 8, 22, 65] exist. This may be because those artists rarely create this type of art, and researchers often follow the artists’ designs. We hope that researchers will not be limited to the scope of artists’ creations. They should try to create 3D visual optical artwork that artists have not explored yet.

Next, we will start with the variables and objectives of the problems introduced in this paper to help researchers understand the research trends and challenges of 3D visual optical art. In Table 2, we summarize the optimization variables and objectives of these algorithms. We find that researchers tend to optimize the shape and color of objects to get the desired appearance. This may be because they chose only to use color as the object’s material to reduce the complexity of the problem. We can use more complex material modeling to create artworks in the future. Furthermore, researchers do not simultaneously use the appearance of the object and the distribution of light on the receiver as objectives. We think this is because their created artworks are of a single type. A feasible way may be combining different 3D visual optical art types to create more attractive works.

**Table 2:** We summarize the papers mentioned in this article for their optimization variables and target

Art type	Reference	Variable			Objective	
		**Shape**	**Color**	**Reflectance**	**Appearance**	**Light distribution**
**Shadow**						
Shadow art	[1, 2, 4, 5, 9, 11, 12, 14]	●				●
	[6, 8, 10]		●		●	
Self-shadowing images	[15, 16]	●			●	
**Reflection**						
Optimizing mirror	[17, 21, 23]	●				●
	[22]	●			●	
Optimizing object	[24, 29–32]	●	●		●	
	[25, 27]	●			●	
Optimizing reflectance	[33–40]			●	●	
**Refraction**						
Caustic	[41, 42, 46, 48–58]	●				●
Volumetric displays	[59–64]		●		●	
Other refraction art	[65, 66]	●	●		●	
	[67]	●				●
	[68]		●			●
**Others**						
Anamorphic objects	[69–71]	●			●	
Illusion objects	[72]	●	●		●	
	[73]	●			●	
Impossible objects	[74, 76–78]	●			●	

Moreover, we also found that only one method [58] chose to apply the neural network to solve the problem. In recent years, many researchers have begun to apply neural networks to the field of visual arts, and the work of Santos et al. [79] introduces them in detail. However, these works are mostly limited to 2D images, using neural networks for object detection, classification, or style transfer of paintings. There are only a few works on how to use neural networks to generate 3D objects [80, 81], but they are far from the level of art.

We believe neural networks have great application potential in 3D visual optics. Although it is difficult to propose an end-to-end neural network algorithm, we can use it as part of the optimization step in the algorithm. For example, using recurrent neural networks (RNNs) to deal with semantics in 3D visual optic arts may be a worthwhile direction to consider. As Su and Kuo [82] mentioned in their article, some works such as [83, 84]have studied the RNNs’ ability to describe the content of images or videos. Maybe the ability of RNNs is slightly insufficient at present, but some works [85, 86] have been proposed by Su and Kuo to improve the RNNs to have stronger learning capabilities, and they also introduced a dimension reduction method [87] for better handle input data. Therefore, we have reason to believe in the potential of RNNs to generate meaningful 3D visual artwork. Another example is shown in Kassubeck et al. [58], who used neural networks in their algorithm to speed up the sampling and gradient computation steps. In addition, how to choose a differentiable renderer based on the type of art is an issue to consider. For shadow art, it may be better to use differentiable rasterization algorithms. For the reflection and refraction art, differentiable ray tracing may be more suitable. If 3D printing technology is used to produce results, the differentiable rendering algorithm should be improved so that a more complex material model can be used to reduce fabrication error. Overall, the combination of deep learning and 3D visual optical art is still a blank, which is worth our research in the future.

In addition, we also found that none of the algorithms proposed by the researchers are generalizable due to the different variables that need to be optimized between different design problems. Although Meyron et al. [54] have successfully solved the caustic design problem of reflection and refraction by using the optimal transport theory, their method still cannot be extended to the entire field of 3D visual art. This leads researchers to lack a general framework to analyze the problem. Sometimes they need to propose a new method to solve a variant of a previous problem.

**Fig. 33:**
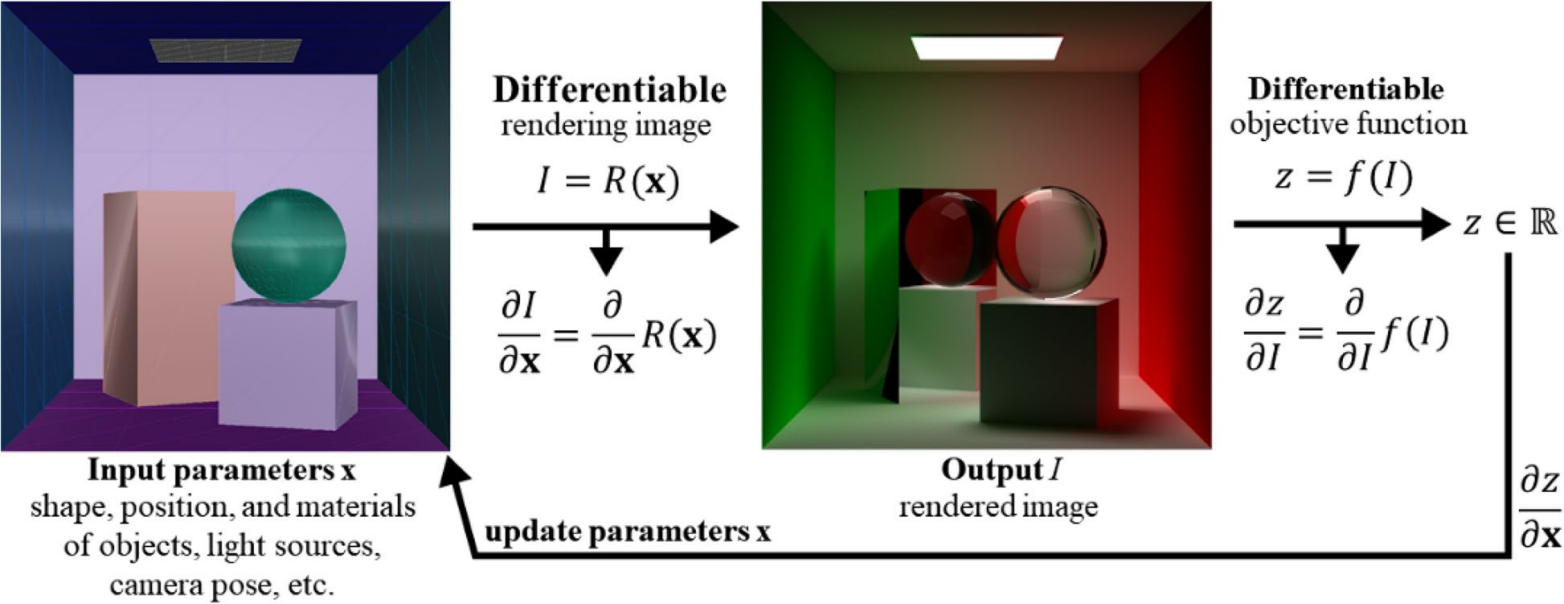
Rendering a scene can be interpreted as a function $R(\mathbf{x})$ where the input is the parameters of the scene, lighting and camera, and the output is the rendered image. Using differentiable rendering allows us to obtain gradient information from the image to the parameters of the scene to apply the gradient descent algorithm to optimize these parameters

The problem mentioned above undoubtedly prevents researchers from further exploration in this field. Inspired by the application of differentiable rendering in the work of Wu et al. [25], we propose a general solution framework to overcome the existing challenges. From Tables 1 and 2, we can conclude that the variables of all 3D visual art design problems are scene parameters, and the optimization objectives are visual effects that can be represented as images. Therefore, all these design problems can be regarded as inverse problems, that is, how to optimize the parameters of the scene according to the error between rendered images and target images. This is often difficult under the standard rendering pipeline because the rendering function is non-differentiable. However, differentiable rendering solves the problem exactly. Using the differentiable rendering technique, we can obtain the gradient from the image to the input parameters, which allows us to update the parameters according to the objective function. Figure 33 shows the process of optimizing parameters using differentiable rendering.

Based on the application of differentiable rendering, we can model any 3D visual optical art design problem as the following optimization problem:

7 $$\begin{array}{cc}\underset{\mathbf{x}}{\mathrm{min}}& {E}_{\text{visual}}\left(\mathbf{x}\right)+w{E}_{\text{constraints}}\left(\mathbf{x}\right)\\ \text{s.t.}& {g}_{i}\left(\mathbf{x}\right)=0,\forall i,\\ & {h}_{j}\left(\mathbf{x}\right)<0,\forall j,\end{array}$$

where $\mathbf{x}$ is the input parameters, ${E}_{\text{visual}}$ is the error between target images and rendered images, ${E}_{\text{constraints}}$ is the soft constraint such as the smoothness term, $w$ is a positive weight, and ${g}_{i}$ and ${h}_{j}$ are the hard constraints such as the maximum displacement of vertices. In our framework, we use a differentiable renderer to render images. Therefore, our objective function is differentiable, and a gradient-based minimization process can be used to solve our problem.

The method of Wu et al. [25] is an example of our idea. Their output mesh is a heightfield with facet colors (Fig. 14). Therefore, in their algorithm, the input parameters are the heightfield and facet colors. The ${E}_{\text{constraints}}$ is the smoothness constraint. They use barrier functions to reformulate their problem to an unconstrained optimization problem. Therefore, some gradient-based algorithms can be applied to solve it.

To further help readers understand our idea, we use our framework to reformulate the three abovementioned methods. The first example is the optimization problem in Baran et al. [10], who used multiple layered occluders to create soft shadow images (Fig. 5). Therefore, the input parameter is the configuration of these occluders. The ${E}_{\text{visual}}$ could be used to ensure the output is connected. With some integer optimization algorithms, we can successfully solve this problem.

Another example is the problem mentioned by Mas et al. [21]. Their algorithm generated a mirror array to create optical art (Fig. 12). The ${E}_{\text{visual}}$ is still the image error. The input parameter is the orientation of the mirror array. The constraint could be a viewpoint stability term, which means we can use an image error between the input image and the image rendered from an offset viewpoint to represent it. Then, the objective function is differentiable and can be solved by many numerical algorithms.

The classical caustic problem can also be solved by using our framework. We can use the lens shapes, represented by triangular meshes, as the input parameters and the smoothness term as the constraint. Once we use differentiable ray tracing to render images, we can easily obtain the gradient from the objective function to the mesh parameters.

Therefore, our framework is general. We hope our framework can help researchers understand and model 3D visual optical art design problems better.

## Conclusions

In this paper, we classify and review the articles about 3D visual optical art design problems in the past decade. Despite the diversity of these design problems, we have grouped them into four major categories based on their corresponding art types. To help researchers quickly learn about the information in this field, we have created two tables based on the characteristics of these 3D visual optical artworks and the variables and objectives of their problems, respectively. We use these two tables to analyze the characteristics of these design problems and the research trends about them. We also find that all these design problems can be reformulated as inverse problems. Therefore, we propose a general solution framework to help researchers better understand and model the 3D visual optical art design problems. Our proposed framework simplifies the 3D visual art design problem, bringing new possibilities to research in this field. As shown in Fig. 33, the input parameters of our framework are not only the shape, color, and reflectivity of the object but all rendering-related parameters, including light sources and cameras. Moreover, we can use more complex models to represent objects, allowing us to simulate the effect of more materials. This means that we do not need to be limited to optimizing the object’s shape, color, etc. Any variable related to the rendering result can be used as an optimized parameter. Moreover, since the problem is simplified in our framework, we can design more complex devices to create visual art. Next, we recommend some future research directions that we think are possible.

### Shadow art

**Fig. 34:**
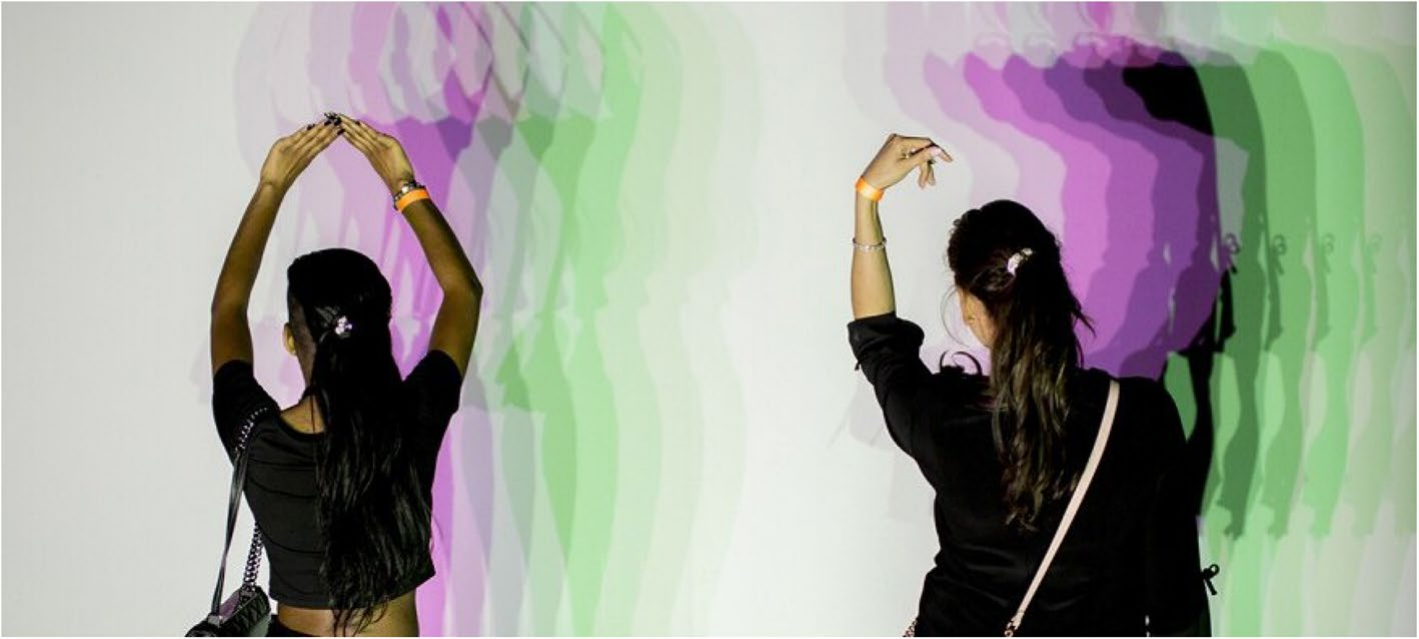
A multiple shadow artwork created by Olafur Eliasson. He used multiple light sources to display the complex visual effects of human shadows

In previous shadow artworks, researchers modified the shape of objects to make shadows under specific lights to form desired patterns. Most of them used a point or directional light to create shadows; thus, their results lack soft shadows and rich detail. Min et al. [9] used an area light, but their results are quite complicated (Fig. 4). In addition, they did not consider the light source as an optimized variable. However, using more complex light sources can make shadow art more interesting (Fig. 34). In the future, we think we can use the shape, position, and even the number of light sources as optimization variables to produce various shadow patterns while using objects with simpler geometries.

### Costume design

**Fig. 35:**
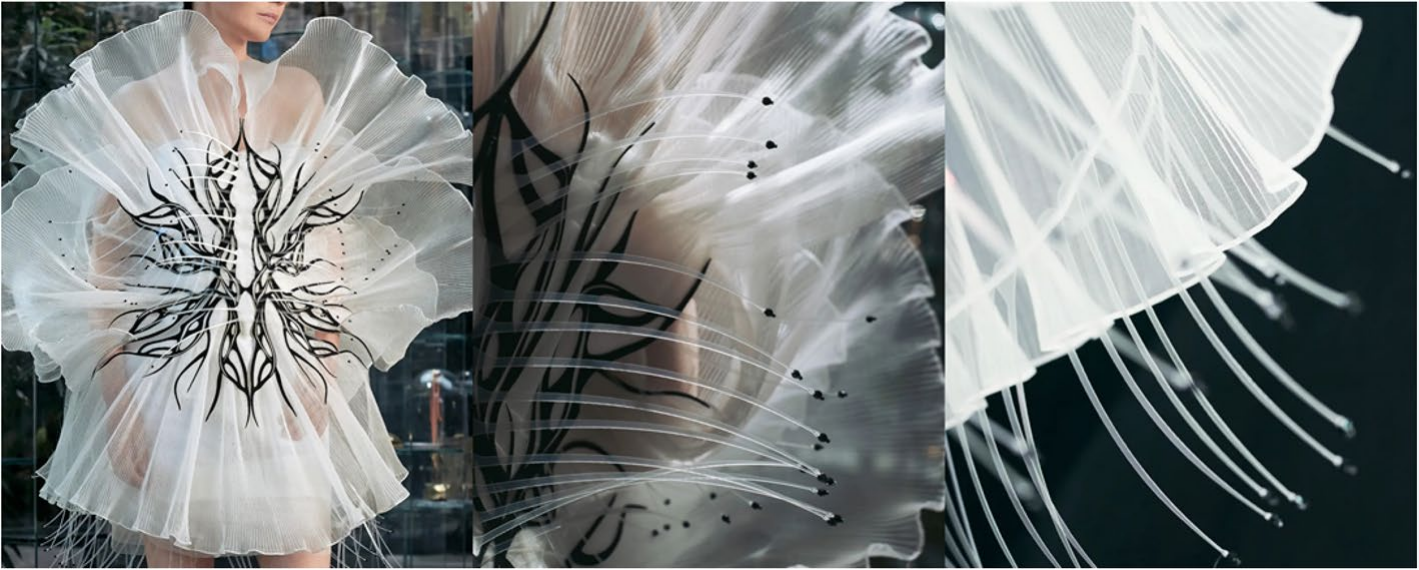
Clothing designed by Iris van Herpen. 3D printing and cloth simulation algorithms help designers create visually stunning garments

The rise of 3D printing technology and cloth simulation algorithms allows more freedom in costume design (Fig. 35). However, the current design process is interactive. The user needs to determine the parameters, perform the simulation to see the result, and then adjust the parameters according to the result. By combining cloth simulation and differentiable rendering, we might be able to make end-to-end design software. The user only needs to input a few images, and the algorithm automatically generates clothing models and fabric parameters that meet the requirements.

### Multiple reflections

**Fig. 36:**
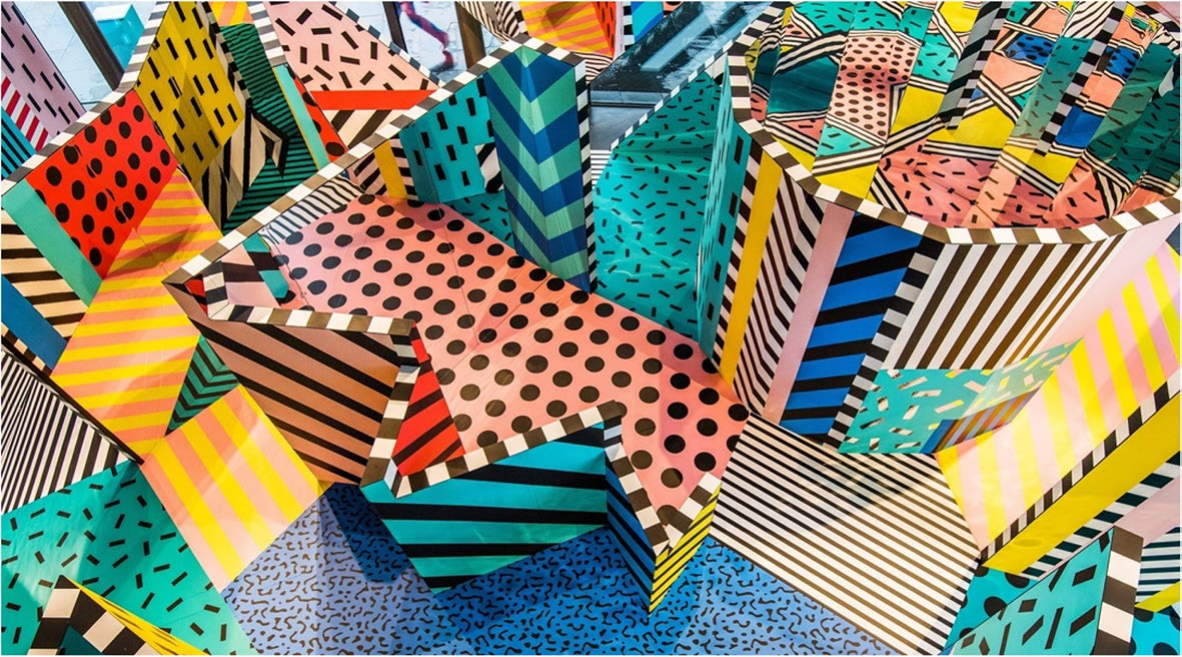
A mirror maze designed by Camille Walala. She used multiple mirrors to create stunning visual art

Adding more mirrors to visual arts is an exciting research direction. Due to the complexity of light path computation, previous works usually only computed one reflection. This limits the design space for the artists to create reflection art. However, nowadays, the existing differentiable ray tracing rendering technology makes it possible to compute multiple reflections. By cleverly placing mirrors so that objects are reflected multiple times between the mirrors, the eye-popping artwork can be presented (Fig. 36).

### Complex material

**Fig. 37:**
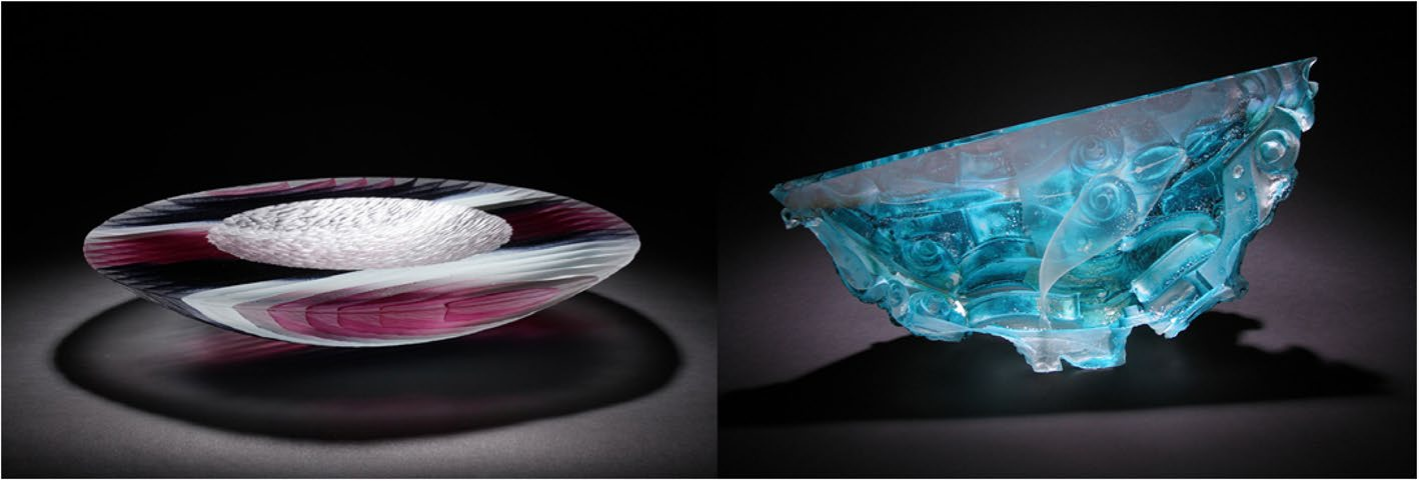
Two artworks created by Colin Reid. He is good at making various works of art using glass

In the previous works, the material modeling of objects was relatively simple. Most of them used color as the material of objects, and in a few other works, they used BRDF. This simplistic approach to modeling limits the expressiveness of their artwork. In contrast, using real-world materials results in far more impressive works (Fig. 37). Therefore, applying a more complex model to represent the material of the object, such as bidirectional scattering surface reflectance distributed function, allows the artist to design better works.

### Fusion of visual arts

**Fig. 38:**
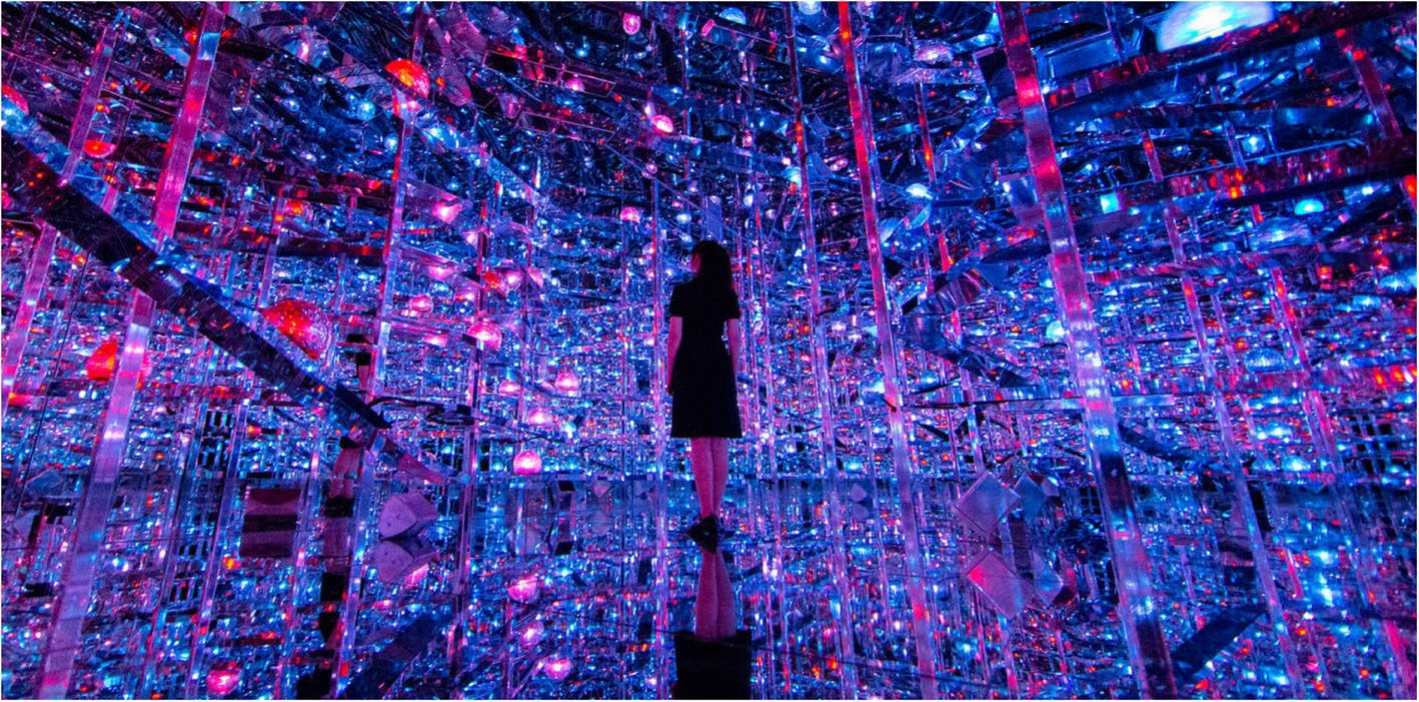
The artwork created by teamLab. They combine multiple art types into one artwork to create a body immersive experience for users

The current research work has only studied a particular type of visual arts. This makes their results simple and incapable of presenting more complex artistic effects. However, more and more artists are now choosing to create complex, large-scale 3D artworks to bring users an immersive visual experience (Fig. 38). We think this is a good research direction for the future. Moreover, our proposed framework unifies various types of visual arts, making it easy to fuse art types. Therefore, researchers can efficiently study various design problems that integrate visual arts under our framework.

### Applications in VR, AR, and interaction areas

**Fig. 39:**
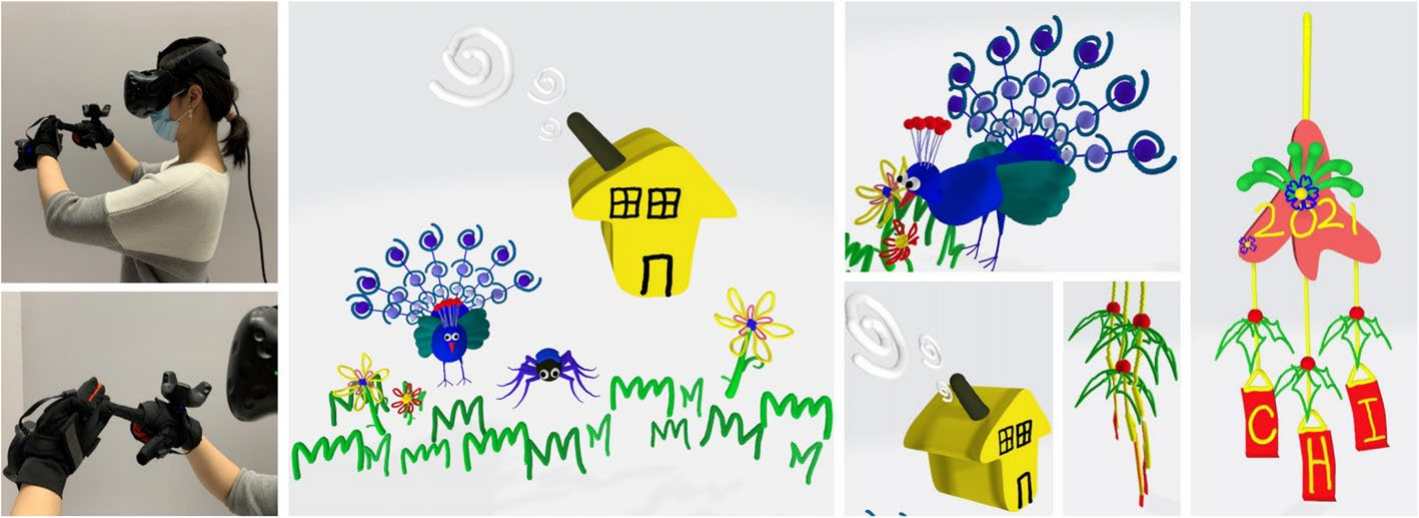
Jiang et al.[88] proposed an algorithm to allow users to draw freely in the virtual world

So far, researchers have focused on fabrication for creating 3D visual optical art in the real world when studying the design problem. However, we can extend it appropriately to integrate with VR and AR technology. For example, Sugiura et al. [33, 34] designed various devices to allow users to paint freely on grass or blankets, whereas Jiang et al. [88] used VR technology to enable people to paint in mid-air freely and intuitively (Fig. 39). We believe there is a correlation between these two works. We can consider reproducing 3D visual optical art in the virtual world. Another direction for future work is to apply the technologies of 3D optical visual art to the field of AR and VR. Arpa et al. [72] studied how to fuse the 2D and 3D parts of a scene; this optical illusion can help us improve user comfort when using AR and VR. Some researchers have explored this aspect. Qian et al. [89] used AR technology to allow users to annotate real-world documents on mobile phones, while Todd et al. [90] studied the impact of hallucinations on interaction in VR. Moreover, since we do not need to consider the fabrication problem, we can use some implicit ways, such as NeRF [91], to represent the object. The implicit function has strong expressiveness when we render images; therefore, it can show richer visual effects. We believe combining 3D visual optical art and the virtual world can improve people’s experience.

Of course, the difficulty of further development in this field is how to properly apply new computer graphics technology to 3D visual art design and create more attractive artwork. However, we believe that as long as we can find the breakthrough and combine it with knowledge in related fields, the development prospect is very broad.

## Data Availability

Not applicable.

## Abbreviations

VC: Visual cryptography
LUT: Look-up table
EVCS: Extended VC scheme
BRDF: Bidirectional reflectance distribution function
CNC: Computer Numerical Control
SoftRas: Soft Rasterizer
UV printer: Ultraviolet printer
SAN: Simulated annealing
SPSA: Simultaneous perturbation stochastic approximation
ROI: Region of interest
TPS: Thin-plate spline
CAGD: Computer aided geometric design
RNN: Recurrent neural network
